# The subventricular zone neurogenic niche provides adult born functional neurons to repair cortical brain injuries in response to diterpenoid therapy

**DOI:** 10.1186/s13287-024-04105-4

**Published:** 2025-01-05

**Authors:** Ricardo Pardillo-Díaz, Patricia Pérez-García, María Ortego-Domínguez, Ricardo Gómez-Oliva, Nora Martínez-Gómez, Samuel Domínguez-García, Francisco García-Cózar, Juan Pedro Muñoz-Miranda, Rosario Hernández-Galán, Livia Carrascal, Carmen Castro, Pedro Nunez-Abades

**Affiliations:** 1 Department of Physiology, University of Seville, Seville, Spain; 2 Division of Physiology, University of Cadiz, Cadiz, Spain; 3 Biomedical Research and Innovation Institute of Cadiz (INiBICA), Cadiz, Spain; 4 Division of Immunology, University of Cadiz, Cadiz, Spain; 5 Department of Organic Chemistry, University of Cadiz, Cadiz, Spain; 6 Biomolecules Institute (INBIO), Puerto Real, Cadiz, Spain; 7 Department of Pharmacology, University of Michigan, Ann Arbor, USA; 8 Department of Neuroscience, Karolinska Institutet, Stockholm, Sweden

**Keywords:** Neural stem cells, Subventricular zone, Neurogenesis, Cortical brain injury, Neuronal differentiation, Newly generated neurons, Morphofunctional properties, Diterpenoids, Brain repair therapy

## Abstract

**Introduction:**

Neural stem cells from the subventricular zone (SVZ) neurogenic niche provide neurons that integrate in the olfactory bulb circuitry. However, in response to cortical injuries, the neurogenic activity of the SVZ is significantly altered, leading to increased number of neuroblasts with a modified migration pattern that leads cells towards the site of injury. Despite the increased neurogenesis and migration, many newly generated neurons fail to survive or functionally integrate into the cortical circuitry. Providing the injured area with the adequate signaling molecules may improve both migration and functional integration of newly generated neurons.

**Methods:**

In here, we have studied the effect of a diterpene with the capacity to induce neuregulin release at promoting neurogenesis in a murine model of cortical brain injury. Using green fluorescent protein expressing vectors we have labeled SVZ cells and have studied the migration of newly generated neuroblasts toward the injury in response the treatment. In addition, using electrophysiological recordings we have studied the differentiation of these neuroblasts into mature neurons and their functional integration into the cortical circuitry. We have studied their electrical properties, their morphology and cortical location.

**Results:**

We have found that EOF2 treatment of adult mice with mechanical cortical injuries facilitates the delivery of neuroblasts into these injuries. The newly generated neurons develop features of fully functional neurons. Our results show that the newly generated neurons receive electrical inputs, fire action potentials, and undergo complete differentiation into neurons recapitulating the stages that distinguish ontogenic differentiation. These neurons develop features representative of neurons belonging the cortical layer in which they are situated. We have also studied that EOF2 facilitates neuregulin release in SVZ cells, a signaling factor that promotes neuronal differentiation. Neuregulin is expressed in microglial cells that reach the injury in response to the damage and its release is increased by EOF2 treatment.

**Conclusion:**

Promoting neuregulin release via diterpene treatment facilitates migration of SVZ-derived neuroblasts to cortical injuries stimulating their differentiation into mature functional neurons, which receive electrical inputs and develop features of cortical neurons. These findings highlight the role of diterpenoids as a potential therapy to repair cortical brain injuries.

**Supplementary Information:**

The online version contains supplementary material available at 10.1186/s13287-024-04105-4.

## Introduction

Cortical injuries, caused by traumatic incidents or neurological disorders, lead to the irreversible loss of neurons and result in significant challenges to cognitive and motor functions. For decades, the adult cortex was considered to have limited regenerative potential. However, recent research has shed light on the role of subventricular zone (SVZ) neurogenesis in cortical injury regeneration [1].

In response to cortical injuries, SVZ neurogenesis plays a crucial role in facilitating regeneration. Injury-induced signals, including growth factors and inflammatory mediators [2], trigger the activation and mobilization of neural stem cells (NSCs) within the SVZ [3]. These activated NSCs give rise to neuroblasts, which physiologically migrate along the rostral migratory stream (RMS) towards the olfactory bulb (OB), where they differentiate into interneurons [4]. However, under certain conditions SVZ generated neuroblasts may alter their migration pattern to contribute to cortical repair and regeneration. Previous reports show that in response to a cortical injury, neural progenitors from the SVZ migrate ectopically to the injured area assisted by blood vessels and reactive astrocytes [5]. Upon reaching the lesion area, inflammatory signaling cues stimulate the differentiation of these progenitors mainly into astrocytes and, very few mature neurons [5, 6]. Thus, neuronal replacement in injuries is a rare event and does not contribute to the recovery of the lost neurophysiological functions probably because the small number of neuroblasts that reach or are generated within the injured area fail to fully differentiate, receive electrical input and incorporate into existing circuits. It is then necessary to develop strategies aimed at enriching injuries with newly generated neuroblasts, which will differentiate into mature, functional neurons.

In the study of neuroblast migration is essential the role of proteins kinases (PK) such as PKA or PKC [7, 8]. In relation to this, previous reports have revealed that the treatment of mouse cortical injuries with a novel PKC activating diterpenoid resulted in neuroblast enrichment and in their differentiation into mature neurons [9], suggesting a role for this molecule at promoting replacement of functional neurons in cortical injuries. The mechanism of action of this diterpenoid is based on its capacity to modify the concentrations of signaling molecules that regulate neurogenesis in response to an injury [9]. Chemotactic and inflammatory cues released by microglial cells and astrocytes seem to play an important role in defining the differentiation of SVZ progenitors into glial cells in response to an injury, in prejudice of neuronal differentiation [10, 11]. In response to an injury, SVZ NSC give rise to a subpopulation of reactive astrocytes in the cortex that contribute to astrogliosis and scar formation [12]. Within the injury environment and the SVZ, growth factors that promote proliferation and glial differentiation are highly expressed such as transforming growth factor alpha (TGFα) [6, 13] and they need to be counterbalanced with signals that promote differentiation such as neuregulins to allow regeneration and replacement of the lost neurons. Interestingly, evidences show that in response to diterpenoid EOF2, which activates novel PKC activity and neuregulin release, these signaling cues [9] may be altered to promote the premature differentiation of neuroblasts and their migration toward the injured area [9] suggesting a role for neuregulin 1 (NRG1) and novel PKC in neuronal replacement in cortical injuries [14, 15].

Despite the evidence showing the activation of SVZ neurogenesis during cortical injury repair and its potential to replace damaged neurons, little is known about whether the generated neuroblasts can differentiate into mature neurons at the injury site and become functional cortical neurons. Earlier studies have focused on this phenomenon by examining the differentiation of neuroblasts within the striatum in response to ischemia [16]. However, further investigations are required to determine the role that SVZ neurogenesis may play in the replacement of functional cortical neurons in injuries.

We have analyzed in here whether the treatment with EOF2, stimulates the maturation and integration of newly generated neurons within the cortical injured tissue. Using ZsGreen fluorescent protein expressing lentiviral vectors, we labeled cells of the SVZ before performing injuries in the primary motor cortex of adult mice. We have studied the migration of ZsGreen-labeled (ZsGreen^+^) neuroblasts toward the perilesional area as well as the time course of the functional differentiation of these newly generated neurons. We have analyzed the functional properties of newly generated neuroblasts and neurons over the course of 7 to 90 days post injury (dpi). Passive and active membrane properties, excitatory inputs and repetitive firing properties were characterized by electrophysiological recordings in ZsGreen^+^ cells and compared with those of unlabeled pyramidal mature neurons of the layer V of the motor cortex. Finally, in order to study the signaling cues responsible for this effect, we have analyzed the time-course of neuregulin expression post-injury in the SVZ and cortex. We show that the expression of NRG1 occurs mainly in microglial cells and to a lesser extent in astrocytes. We have facilitated NRG1 release using pharmacological compound EOF2, which in addition stimulates neuroblast differentiation, on the generation of new neurons in response to a mechanical cortical brain injury.

## Methods

### Animal subjects

CD1 mice of both sexes were used throughout this study. Animals were housed under controlled conditions of temperature (21–23 °C) and light (LD 12:12) with free access to food (AO4 standard maintenance diet, SAFE, Épinay-sur-Orge, France) and water. Care and handling of animals were performed according to the Guidelines of the European Union Council (2010/63/EU), and the Spanish regulations (65/2012 and RD53/2013) for the use of laboratory animals.

The number of animals used in each experiment was determined based on previous studies [17–19]. Adult male mice were randomized during the first week after birth by cross-fostering and used when they became two months old. The protocol used has been authorized by the Ethics Committee of the “Consejería de Agricultura, Ganadería, Pesca y Desarrollo Sostenible de la Junta de Andalucía”, in Spain with the protocol entitled “Neural Replacement thErapies in two Models of brain damage: towards fInnding New Drugs (REMIND)” approval number 04/03/2020/033. All studies involving animals are reported in accordance with the ARRIVE guidelines 2.0 for reporting experiments involving animals [20, 21]. The number of animals used in each experiment is indicated in the figure legends.

### Design of the study

Mice used in the study were lesioned in the primary motor cortex by generating controlled mechanical injuries while anesthetized by a cocktail of ketamine (100 mg/kg) and xylazine (20 mg/kg). For the migration studies, mice were injected with lentiviral vectors prior to the injury in the same surgical act. Once injured, vehicle consisting on saline solution was used in control animals and diterpene EOF2 was used as treatment. Either vehicle or EOF2 were administered daily by intranasal infusions for either 14, 28, or 56 days as we describe in the paragraphs below. Upon the completion of the treatments, mice were anesthetized with a cocktail of ketamine (100 mg/kg) and xylazine (20 mg/kg) and cerebrospinal fluid (CSF) was extracted as explained below, then a dose of Dolethal® (Ventoquinol, Lure, France) containing a lethal 50 mg dose of pentobarbital to euthanized the animals was applied followed by either brain perfusion with paraformaldehyde (for histological studies) or brain extraction (for molecular biology and studies). See description of the different procedures below. In the case of electrophysiological studies mice were anesthetized with a lethal dose of anesthetic previous to perfusion with artificial CSF.

In all in vivo experiments, the experimental groups used were saline treated (control) or EOF2 treated mice (EOF2). In postmortem studies, the experimental unit was the single animal and sample size for each experiment is indicated in the figure legends. In electrophysiological studies the experimental unit was the single cell and the sample size is indicated in Additonal file 1: Tables S4-S7.

### Injection of ZsGreen expressing lentiviral vectors in the SVZ and unilateral mechanical cortical brain lesions

Controlled unilateral mechanical cortical brain injuries were performed in the primary motor cortex of the right brain hemisphere of anesthetized mice. Mice were anesthetized using an anesthetic mixture composed of ketamine (100 mg/mL) and xylazine (20 mg/Kg) in sterile physiological saline. Using a stereotaxic frame (Harvard Apparatus), a longitudinal incision was made in the skin of the head to expose the skull and proceed to inject the ZsGreen expressing lentiviral vector. At Bregma/−0.8 mm, a small craniotomy was performed using a hand drill and a 0.9 mm drill bit (Meisinger, Neuss, Germany). Then a Hamilton Gastight syringe (Hamilton Company, NV, USA) was introduced attached to the stereotaxic device through the incision made, and 1 µL of the ZsGreen virus was injected into the lateral ventricle at a rate of 0.1 µL/min. The lentiviral construct was produced by us as described previously [14]. HEK Lenti-XTM 293 T were used as packaging cell lines to produce lentiviral supernatant as previously described [22]. Cells were co-transfected with the pHRSincPPT- SEW transfer vector expressing the green fluorescent protein Zs-Green, together with plasmids pCMV∆R8.91, coding for HIV-1 GAG / POL proteins and pMD2.G for pseudotyping with the Vesicular Stomatitis Virus G protein (VSVG). Cells were transfected in OptiMEM™ medium (Thermo Fisher Scientific Inc., Carlsbad, CA, USA) by polyethylenimine (PEI)-mediated transfection [23] and after one hour the medium was replaced by DMEM supplemented with 10% fetal calf serum (GIBCO; www.thermofisher.com/gibco). Supernatants were collected at 48 and 72 h, centrifuged at 2100 g for 5 min to remove cell debris and subjected to two concentration rounds using Lenti-X™ Concentrator (Clontech;Mountain View, CA, USA) to obtain a clean high-titer virus-containing pellet. Briefly, viral supernatants were incubated for 30 min at 4 °C with 3 volumes of Lenti-X concentrator reagent, centrifuged at 1500 × g for 50 min and the pellet resuspended in 1 mL PBS. Lenti-X was further added and upon 30 min incubation at 4ºC and an additional centrifugation, the pellet was snap frozen in liquid nitrogen and stored at −80 °C until use. Viral titers were determined, by evaluating their efficiency in transducing Jurkat cells by means of a Cyto-flex™ flow cytometer (Beckman, Indianapolis, IN) 48 h after transduction. Viral titers were always above 2 × 10^5^ transducing units (TU) per mL.

In the same surgical act, mice were unilaterally lesioned in the right hemisphere of the primary motor cortex. They were craniotomized with a manual drill at +1.5 mm rostral and -1.1 mm lateral to Bregma. Thereafter, a controlled mechanical lesion was performed in the underlying primary motor cortex using a manual drill (0.9 mm diameter). This drill was allowed to penetrate 1 mm below the bone surface. Mice were injured and placed into a controlled cage during the required dpi that depended on the treatment and experimental design. Lesions were performed unilaterally; the injured hemisphere was considered the ipsilateral side, while the intact hemisphere was considered the contralateral hemisphere and was used as a control. After performing the injury, craniotomies were sealed by surgical cement (Fisher Scientific) and the incision made in the skin was sutured. Subsequent analgesic and aseptic measures were taken to ensure animals welfare. This procedure was previously stablished by our research group and has been used elsewhere [6, 9, 14, 24].

### Intranasal administration of EOF2

EOF2 (CAS number 2230806–06–9) was produced by us as previously described [9] and was delivered intranasally while the animal was placed in a standing position with an extended neck as previously described [25]. Eighteen microliters of each solution (5 μM EOF2 in saline, or saline as vehicle) was delivered over both nasal cavities alternating 3 μL/each using a micropipette. Mouse was maintained in this position for 10 additional seconds to ensure all fluid was inhaled. In all experiments, mice were coded and treatment (vehicle or EOF2) was assigned randomly to code numbers and applied. The treatment was administered daily until 90 dpi.

### Brain processing for immunohistochemistry studies

At the end of the treatment, brains were perfused with paraformaldehyde (PFA) and sliced using a cryotome into 30 μm sections. Immunohistochemistry was performed as previously described [18, 24, 26]. See antibodies in Additonal file 1: tables S1-S3. The markers used to detect the different cell types were doublecortin (DCX) to detect neuroblasts, glial fibrillary acidic protein (GFAP) to detect astrocytes, ionized calcium binding adaptor molecule 1 (Iba1) to detect microglial cells, neuroepithelial stem cell protein (nestin) to detect neural stem cells and progenitor cells and the neuronal nuclear protein (NeuN) to detect mature neurons.

### Brain slices obtention for electrophysiological studies

Brain slices were acquired from previously treated mice by anesthetizing them and perfusing with a modified artificial CSF (ACSF) or cutting solution. Following perfusion, the brain was swiftly extracted, and after removal of the cerebellum and rostral telencephalon, coronal slices of 300 µm thickness were obtained using a vibratome (Leica VT1000S, Leica Biosystems, United Kingdom). These slices were then incubated in a chamber containing cutting solution at 34º C for 10 min, followed by transfer to another chamber filled with recording solution at room temperature for at least 1 h before further use. The composition of the different ACSF used was as follows (data in mM): i) Recording solution: 126 NaCl, 2 KCl, 1.25 NaH_2_PO_4_, 26 NaHCO_3_, 10 glucose, 2 MgCl_2_, and 2 CaCl_2_; ii) Cutting solution: 92 NMDG, 2.5 KCl, 1.2 NaH_2_PO_4_, 30 NaHCO_3_, 20 HEPES, 25 Glucose, 4 MgCl_2_, 0.1 CaCl_2_. Both solutions were bubbled with 95% O_2_–5% CO_2_ (pH 7.4, adjusted with HCl; 295–305 mOsmol/kg).

### Whole-Cell patch clamp recordings and analysis

After one hour of incubation in the holding chamber, the slices were transferred to the recording chamber of the microscope. To visualize the cells, a Nikon Eclipse FN1 microscope equipped with infrared differential interference contrast (IR-DIC) optics, a 40 × water immersion objective and an infrared camera WAT-902H2 is used. In the holding chamber, the slices were constantly perfused with ACSF aerated at room temperature and at a rate of 1 mL/min using a peristaltic pump (Harvard Apparatus MPII, Holliston, MA, USA). The micropipettes used to perform the patch-clamp were obtained from borosilicate glass capillaries (od 1 mm, id 0.58 mm, length 10 cm, Sutter) stretched with a vertical puller (PC-10, Narishige, Tokyo, Japan) adjusted to get a resistance between 3 and 6 MΩ. The micropipettes were filled with a K-gluconate based solution with the following composition (in mM): 120 K-Gluconate, 10 KCl, 10 phosphocreatine disodium salt, 2 Mg-ATP, 0.3 Na-GTP, 0.1 EGTA, 10 HEPES. pH was adjusted to 7.3 using KOH and the osmolality to 285 mOsmol/kg with sucrose with the help of an osmometer (Osmomat 300, gonotec). To perform the recordings, the micropipettes were placed using a micromanipulator (MP-225, Sutter Instrument, CA, United States) in the injured area. In this area, ZsGreen labelled cells were identified using the fluorescence microscopy system coupled to the Nikon microscope (see Additonal file 1: Fig. S1A). To achieve the whole-cell patch-clamp configuration, we use an amplifier (MultiClamp 700B) and a Digidata 1550 analog-to-digital converter (Axon Instruments, Molecular Devices, Sunnyvale, CA, United States). Recordings were acquired with the pCLAMP 10.4 software (Molecular Devices), lowpass Bessel-filtered at 3 kHz and the data were digitized at 20 kHz. For the data analysis, Clampfit 10.4 software (Molecular Devices). Series resistances was typically 10–20 MΩ, and the experiments were discarded if higher than 25 MΩ. Liquid junction potentials were compensated automatically.

### Current clamp studies

In this study, both passive and active membrane properties of the cells were studied as previously described [27, 28]. In brief, the resting membrane potential was calculated by subtracting intracellular from extracellular potential after the removal of the recording electrode. Input resistance was measured through the injection of hyperpolarizing and depolarizing square current pulses (500 ms, 1 Hz) with 10 pA increments between each one, and then calculated as the slope of the current–voltage relationship, following Ohm's Law. Rheobase, defined as the minimum intensity of current necessary to provoke an action potential, was determined by applying square pulses of 100 ms, 1 Hz, with 10 pA increments. Voltage threshold and depolarization voltage were computed relative to the resting membrane potential. To ascertain the spike threshold, action potential recordings were differentiated, with the spike onset identified as the membrane potential at which the first derivative exceeded 10 V/s [29, 30].

Action potential amplitude and duration were calculated based on peak voltage and width at half amplitude. Repetitive firing properties were assessed by applying depolarizing current steps (1 s, 0.5 Hz) with 20–50 pA increments. Maximum firing frequency was defined as the highest number of spikes achieved during repetitive discharge, regardless of current intensity, while frequency gain was determined as the slope of the relationship between firing frequency and applied current. Cancellation current represented the intensity at which the neuron ceased firing during maximal discharge.

### Voltage clamp studies

Voltage-dependent currents were elicited by 50 ms square depolarizing pulses ranging from −60 to + 40 mV, in 10 mV steps. No leak substraction was performed. Current amplitudes were measured in the peak for inward currents and at the end of the pulse for outward currents. Conductances were calculated as chord conductance [31]. Thus, G = I/(V-V_E_), being G, conductance, I, the measured current, V, command voltage and V_E_ the theorical Nernst potential for potassium (outward currents) or sodium (inward currents). Tetrodotoxin (TTX) (Tocris), Tetraethylammonium (TEA), (Sigma-Aldrich) and 4-AP (Sigma-Aldrich) were diluted in the bath solution for blocking the different conductances.

To investigate spontaneous postsynaptic currents (sPSC), 60 s continuous recordings were conducted following previously described methods [32]. The holding potential was clamped at 0 mV for spontaneous inhibitory postsynaptic currents (sIPSCs) or −60 mV for spontaneous excitatory postsynaptic currents (sEPSCs). Synaptic events were detected and analyzed using EasyElectrophysiology software. A template was created by fitting a function to a single event, and subsequent events were extracted using varying detection thresholds. False positives were manually removed, and real events were fitted with a biexponential function to determine percentage appearance, frequency (events per minute), and amplitude (baseline to peak). To assess the nature of synaptic events, CNQX (50 μM), APV (20 μM), and SR95531 (gabazine, 20 μM) were used, all of which were purchased from Tocris. The protocol involved initially superfusing each slice with normal ACSF for control recordings, followed by ACSF containing the drugs to record voltage responses.

### Morphological study of the newly generated neurons

To carry out the morphometric study of the new generated cells, dye-filling technique was used. For this purpose, iontophoretic injection of 0.2% neurobiotin (Vector Laboratories, Burlingame, CA, USA) contained in the internal pipette solution was carried out by applying current steps of 400 pA of 500 ms at 0.5 Hz for 20 min [33]. Slices containing labeled cells were deposited in a 4% paraformaldehyde solution at 4º C overnight and then transferred to 30% sucrose in phosphate buffer at 4º C to maintain them. Thereafter, dye-filled neurons were revealed with a goat-polyclonal antibiotin Texas Red conjugated antibody (1:900) from Rockland (Pennsylvania, USA). A Zeiss LSM 900 Airyscan 2 confocal microscope was used for the visualization and reconstruction of the labeled cells. Stacks of 30–70 photographs were performed, using a 1 µm interval. Images were processed with ZEN 3.2 Blue Edition software and the cells were reconstructed using the Neurolucida 360 version 2020 3.1 system (MicroBrightField, Williston, VT, United States).

Analysis were made according to previous works [33, 34]. The quantitative morphometric data presented in this study were generated using Neuroexplorer software. Each dendritic segment was systematically assigned an order in a centrifugal manner, from the soma to the terminal segments (see Additonal file 1: Fig. S1). A node, or intersection, was defined as any bifurcation along the dendrite. Furthermore, a segment represented the portion of the dendrite linking either the soma to an intersection or two intersections, while a terminal segment denoted the part connecting the last branching point to the dendrite's terminal ending. This study focused on three key aspects of neurons. Firstly, neuron surface area was examined, with total dendritic surface area defined as the sum of each dendrite's surface area and total surface area calculated as the sum of somatic and dendritic areas. Secondly, dendritic length was investigated, specifically targeting total dendritic length, which encompasses the sum of individual dendrite lengths. Lastly, neuronal complexity was analyzed. Branch order was scored to measure dendritic complexity, representing the highest order reached in each neuron. Additional complexity measures included the total number of segments, representing the entirety of segments within a neuron; the number of nodes, indicating the number of intersections; and the number of terminals, defined as the sum of terminal segments within a neuron. Sholl diagrams were constructed for the reconstructed cells. Neurons were oriented along dorsal and lateral axes, with the soma positioned at the center of concentric circles progressively increasing by 50 µm in radius. The number of dendrites intersecting each circle was recorded as part of the analysis.

The experimental animal groups for electrophysiological recordings and morphometric analysis were as follows: Group 1 consisted of animals analyzed at 7–14 dpi, Group 2 at 15–28 dpi, Group 3 at 29–56 dpi, Group 4 at 57–90 dpi, and Group 5, or control group, comprised pyramidal neurons from layer V recorded from the contralateral side to the injury in 3-month-old animals.

### RNA isolation, reverse transcription and real-time quantitative PCR

For RT-qPCR analysis, RNA was isolated from the SVZ; intact SVZ were processed for RNA extraction using the TRIzol (Cat. 15,596,026, Invitrogen, Carlsbad, CA, USA), separation method, following the manufacturer’s instructions and resuspended in purified nuclease-free water. RNA was quantified using a BioTek’s Synergy Mx fluorimeter (BioTek Instruments, Inc, Winooski, VT, USA). cDNA was prepared from 500 ng RNA using iScriptTM cDNA Synthesis Kit (Cat.1708890, Bio-Rad Laboratories Inc, Hercules, CA, USA) on a Techne Genius thermal cycler (Techne Ltd., Cambridge, UK). The 15 μl RT-qPCR reaction mix contained 7.5 μl 2X iTaq Universal SYBR Green Supermix (Cat. 1,725,122, Bio-Rad Laboratories Inc, Hercules, CA, USA), 10 nmol of both the forward and the reverse primers, and 1 μl of the sample. The PCR thermal profile included 40 cycles of denaturation at 95 °C for 10 s, an annealing temperature according to each set of primers for 15 s, and extension at 72 °C for 20 s, followed by a melting curve analysis. Each sample was analyzed in triplicate. The mRNA level of rRNA18S was used as internal control. Relative quantification values of mRNA expression were calculated as 2 (Livak Method). Oligonucleotides used in this study were designed by BLAST and were obtained from Merck (Madrid, Spain). Primer sequences (5 ´−3 ´) for detecting expression of mouse mRNA were the following: for NRG1, FW: CGCTGTTCTGGTCTCATCCG, RW: GCGGTGGAGTGGAGTGTAAG; for ErbB4, FW: TACCTCCTCCCATCTACACATCC, RW: CCTCTGGTATGGTGCTGGTTG; for PKCδ FW: GAGGCCTTGAACCAAGTGACCC and RW: CTTGCCA TAGGTCCAGTTGTTG.

### PKC kinase activity assay

Mice were injured as previously described and sacrificed 7, and 14 dpi. Then brains were removed and the tissue corresponding to the SVZ and the perilesional cortex area was used in the assay. Tissue was mechanically disaggregated and homogenized in PBS buffer followed by a step of ultrasound treatment and the homogenate centrifuged 10,000 × g for 15 min. Then, the protein content was measure in the homogenates using the BCA method (ThermoFisher Scientific, Rockford, IL, USA), and 1.5 μg of crude protein of each homogenate was used per assay. The amount of PKC kinase activity was measured in each sample using the PKC Kinase Activity Assay Kit (Abcam, Cambridge, U.K.; cat. No. ab139437), following the manufacturer’s instructions. Positive controls (20–60 ng of purified active PKC supplied by the kit) and blanks (diluent only) were included in each independent determination. Blanks were subtracted from measurements before comparisons were made. Activity was calculated related to the value of the contralateral side.

### SVZ cell isolation and culture

NPCs were obtained from the SVZ of 7-day postnatal mice following the same procedure described in [19]. Neurosphere cultures were maintained in defined medium (DM) composed of Dulbecco’s modified Eagle’s medium/F12 medium (1:1 vol/vol) plus 1 mg/L gentamicin (GIBCO) and the B27 supplement (Invitrogen, Carlsbad, CA). EGF (20 ng/mL) and bFGF (10 ng/mL; both from PeproTech, Frankfurt, Germany) were added to DM for culture expansion.

### Neurosphere differentiation assay

Neurosphere cells obtained from cultures that had gone through three consecutive passages were centrifuged, resuspended in defined medium without growth factors, and seeded. NRG1 soluble ligand (R&D Systems) was added at different concentrations (1, 5 or 10 ng/mL) and cells were maintained for 72 h before being fixed for immunocytochemistry. The different cells phenotypes obtained as a result of differentiation were detected by the presence of the marker beta-III-tubulin (neurons and neuroblasts) or GFAP (astrocytes). Each experiment was performed using triplicate samples. The measurements are the average of three independent experiments. The in vitro model to study differentiation had been successfully used in previous reports [35–37].

### Cloning of human TGFα and NRG1 cDNA fused to eGFP and mCherry

Full-length cDNA encoding the membrane-bound isoform of human pro-neuregulin-1 β1-type (NRG1, NCBI reference sequence: NP_039250.2) with mCherry cDNA inserted between nucleotides 93 and 94 of NRG1 open reading frame was cloned into pEGFP-N1 to add EGFP cDNA to the 3′ end. Construct was synthesized by GeneCust (Boynes, France) to generate the mCherry-NRG1-GFP construct.

### Time-lapse experiments and fluorescence analysis of recombinant mCherry-NRG1-eGFP protein in the culture medium of NPC

NPC were plated in μ–dishes (35 mm high; Ibidi) and transfected with mCherry-NRG1-eGFP construct. After overnight incubation, cells were left for 30 min in serum-free Fluorobrite DMEM (Thermo Fisher Scientific) and used either in time-lapse experiments. Cells were treated with EOF2 compound (5 µM) and images were taken every 2 min. Images of 10 independent cells per condition were analyzed. Measurements are the average of three independent experiments.

### HEK293 culture, cloning and transfection

HEK293T obtained from ATCC (Manassas, VA, USA) were cultured and transfected as previously decribed [14]. After an overnight incubation, cells were left for 30 min in serum-free Fluorobrite DMEM (Thermo Fisher Scientific) and used either in fluorescence experiments.

### Fluorescence analysis of mCherry-fused NRG1 in the culture medium of HEK293

HEK293T were plated in µ–dishes (35 mm high, Ibidi, Munich, Germany). Cells were treated with EOF2 for 30 or 180 min. For fluorescence measurements, 200,000 HEK293T cells were plated in 1 mL of medium Costar® 12-well cell culture microplate and fluorescence in the culture medium was measured as described in the legend of figure 10.

### Statistical analysis

The data and statistical analysis comply with the recommendations on experimental design and analysis in pharmacology [38]. All statistical analyses were conducted on raw data. Results are presented as the mean ± standard error of the mean (SEM), where 'n' denotes the number of cells or animals included. Statistical calculations were performed using GraphPad Prism software. Initially, the normality of the data distribution was assessed using the Shapiro–Wilk test. For comparisons of means between groups, a repeated measures analysis of variance (ANOVA) was applied. If significant differences were detected, the Tukey test was used for pairwise comparisons between groups. When comparing only two unpaired experimental groups, the Student's t-test was employed. To determine statistically significant differences in the percentage of cells firing action potentials, repetitive discharge, or synaptic inputs between groups, the Chi-square test for independence was used. If the expected frequencies were small, Fisher's exact test was utilized. A 95% confidence interval was applied in all analyses, and groups were considered statistically different if p ≤ 0.05. Unless indicated, in all figures and in tables included in Additonal file 1, asterisk (*) indicates statistical differences between groups, and crosses (†) indicate differences between the various groups and the 57–90 dpi group.

## Results

To assess the ability of EOF2 to enhance neuroblast enrichment in brain injuries and their differentiation into mature neurons, lentiviral vectors expressing ZsGreen were injected into the lateral ventricle of adult mice, followed by a controlled motor cortex injury. Mice received intranasal EOF2 or saline (control) for 3, 14, or 28 days post-injury, and the SVZ and perilesional areas were analyzed for newly generated neuroblasts, identified as DCX^+^ cells expressing ZsGreen.

### Neuroblast enrichment in mechanical cortical injuries treated with EOF2

**Fig. 1:**
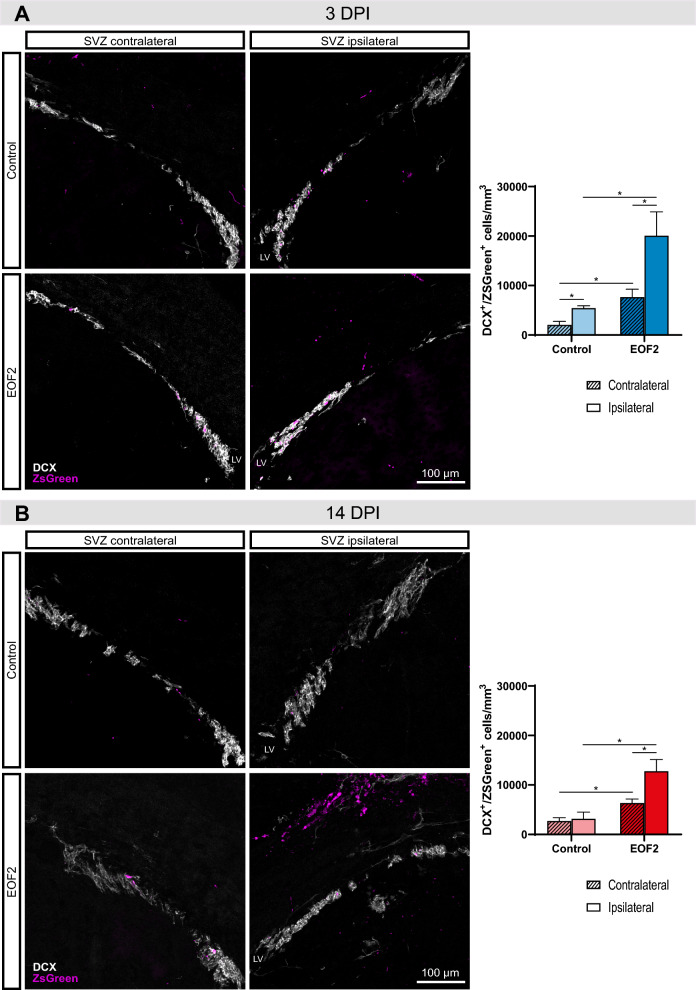
The SVZ responds to a cortical injury by increasing DCX^+^/ZsGreen^+^ and this response is enhanced by the EOF2 treatment. A lentiviral vector expressing ZsGreen was injected in the lateral ventricle ipsilateral to the lesion to mark SVZ cells in the same surgical procedure of the injury. Immunohistochemistry was performed for the detection of the neuroblast marker DCX and ZsGreen (shown in magenta to enhance its visibility). LV = Lateral Ventricle; scale bars represent 100 µm. **A** (Left) Representative confocal images of the subventricular zone (SVZ) of adult mice 3 days post-injury (dpi), treated intranasally with vehicle (upper panel) or EOF2 (lower panel). (Right) Quantification of DCX^+^/ZsGreen^+^ cells/mm^3^ in the contralateral and ipsilateral SVZ related to a cortical brain injury in mice treated with EOF2 or vehicle (control) 3 dpi. Data show the mean ± SEM of 6 animals per group. Statistical analysis: * p = 0.0007 control contralateral vs. control ipsilateral; p = 0.0301 EOF2 contralateral vs. EOF2 ipsilateral; p = 0.0003 control ipsilateral vs. EOF2 ipsilateral; p = 0.0045 control contralateral vs. EOF2 contralateral in two-way ANOVA.** B** (Left) Representative confocal images of the subventricular zone (SVZ) of adult mice 14 days post-injury (dpi), treated intranasally with vehicle (upper panel) or EOF2 (lower panel). (Right) Quantification of DCX^+^/ZsGreen^+^ cells/mm^3^ in the contralateral and ipsilateral SVZ related to a cortical brain injury in mice treated with vehicle (control) or EOF2 14 dpi. Data show the mean ± SEM. Statistical analysis: * p = 0.0049 control contralateral vs. EOF2 contralateral; p = 0.0228 EOF2 contralateral vs. EOF2 ipsilateral; p = 0.0172 control ipsilateral vs. EOF2 ipsilateral in two-way ANOVA

As shown in Fig. 1, in control animals at 3 dpi (Fig. 1 A) the number of DCX^+^ neuroblasts that had incorporated ZsGreen in the SVZ was higher in the ipsilateral SVZ than in the contralateral SVZ. Interestingly, treatment of mice with EOF2 increased the total number of neuroblasts in both the ipsilateral and the contralateral SVZ in comparison with the control. Also, the number of ZsGreen^+^ neuroblasts in the ipsilateral SVZ of EOF2 treated mice was higher than in the contralateral SVZ. However, at 14 dpi (Fig. 1 B), the difference in the number of ZsGreen^+^/DCX^+^ cells in the ipsilateral SVZ of control animals was not observed whereas this difference was observed in EOF2 treated mice, in which a higher number of ZsGreen^+^/DCX^+^ cells was observed in both the ipsilateral SVZ and the contralateral compared to control mice. These results indicated that in response to the injury the SVZ incremented the number of neuroblasts and the treatment with EOF2 magnified this response.

### Migration of SVZ neuroblasts towards the injury stimulated by EOF2

In order to study whether EOF2 treatment exerted an effect on the migration of neuroblasts from the SVZ to the OB in injured mice, we also analyzed the number of neuroblasts found in the OB that were labeled with ZsGreen. As shown in Additonal file 1: Fig. S2, at 14 dpi, the number of ZsGreen^+^/DCX^+^ neuroblasts found in the ipsilateral OB core of EOF2 treated mice was higher than that of the contralateral side (Additonal file 1: Fig. S2 D-d, G). Such difference was not observed in control mice (Additonal file 1: Fig. S2 B, G). At 28 dpi, the number of ZsGreen^+^/DCX^+^ neuroblasts found in the ipsilateral OB core of EOF2 treated mice was still higher than that of the contralateral side and higher than that of the ipsilateral OB core of control mice (Additonal file 1: Fig. S2 C, E, G). Identical differences were observed in the granular cell layer (Additonal file 1: Fig. S2 A, F). These results indicated that no effect of the injury on neuroblast migration to the OB was observed neither at 14 nor at 28 dpi. However, in the presence of EOF2, migration of neuroblasts to the OB is facilitated.

**Fig. 2:**
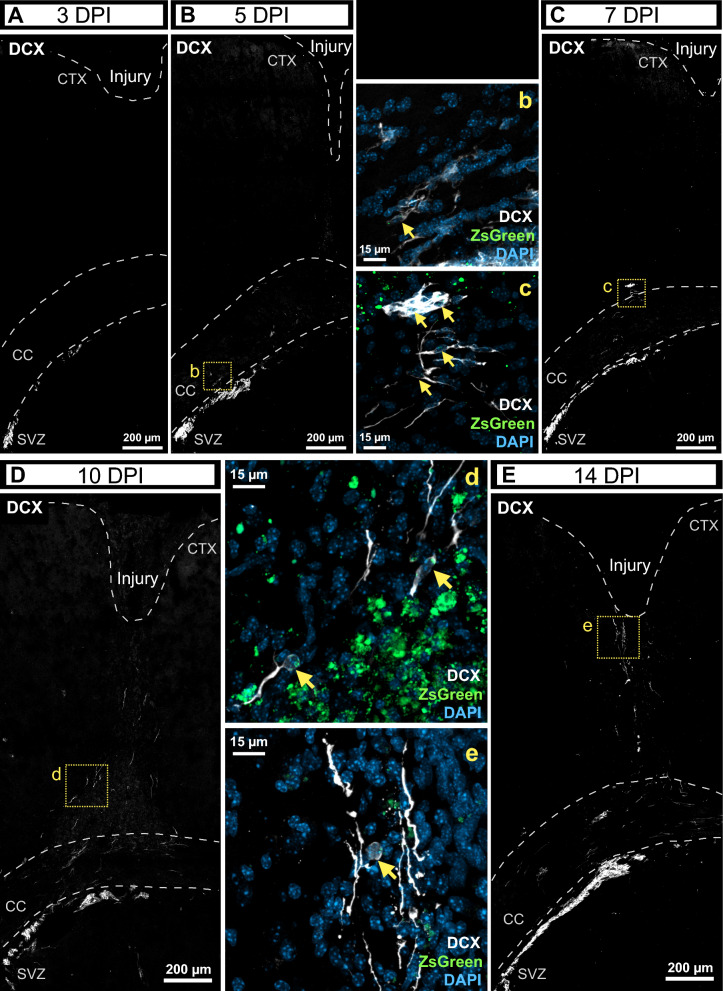
EOF2 administration promotes migration of neuroblast from the SVZ to cortical injury within 14 days. Mice were mechanically injured in the primary motor cortex. A lentiviral vector expressing ZsGreen was injected in the lateral ventricle ipsilateral to the lesion to mark subventricular zone (SVZ) cells in the same surgical procedure of the injury. EOF2 was intranasally administered from the day of the injury until sacrifice. **A-E** Representative confocal images showing migration of neuroblast through immunodetection of DCX at 3 days post injury (dpi) (A), 5 dpi (B), 7 dpi (C), 10 dpi (D) and 14 dpi (E). Panels A-E only display DCX^+^ labeling to highlight the presence migrating neuroblasts. CTX = cortex; CC = corpus callosum; SVZ = subventricular zone. Dotted white lines indicates the limit of the injury and CC; dotted yellow lines indicate the site of magnifications. b-d. Magnifications of neuroblasts (DCX^+^ cells) expressing ZsGreen and migrating to the injured site at 5 dpi (b), 7 dpi (c), 10 dpi (d) and 14 dpi (e). Yellow arrows indicate colocalization of the neuroblast marker DCX, nuclear marker DAPI and ZsGreen. 6 animals were used per group

Previous studies have shown that neuroblasts indeed alter their migration patterns and move towards areas of cortical injury. In response to traumatic brain injuries, neuroblasts migrate from the SVZ to the injury site, influenced by various signaling molecules and environmental cues ​[39]​. Additionally, it has been observed that neuroblasts move toward brain lesions in different models of cortical injury, supporting the idea of altered migration patterns in response to such injuries​ [13]. Thus, in light of the results that indicated a facilitation of neuroblast migration induced by EOF2, it was next analyzed whether migration of neuroblasts to the perilesional area was also facilitated by the treatment. Mice were given BrdU injections for three consecutive days. Then, three days after the last dose of BrdU, an intracerebroventricular injection of the ZsGreen lentiviral vector was given to each mouse before injuries were performed as indicated above. Mice were then treated for 1, 3, 5, 7, 10 and 14 days with EOF2 and sacrificed on the last day of treatment. In these mice it was analyzed the presence of ZsGreen^+^/DCX^+^ (Fig. 2) and BrdU^+^/DCX^+^ (Additonal file 1: Fig. S3) neuroblasts in the area included between the SVZ, and the injured cortex. As shown in Fig. 2, no DCX^+^ cells are observed leaving the SVZ at 3 dpi (Fig. 2 A), whereas as soon as 5 dpi DCX/ZsGreen^+^ cells can be observed leaving the SVZ and crossing the corpus callosum toward the injury (Fig. 2 B,b). Then at 7 dpi DCX^+^/ZsGreen^+^ cells were identified that had crossed the corpus callosum and were located close to the injury (Fig. 2 C,c). The number of DCX^+^/ZsGreen^+^ cells found in the area between the corpus callosum and the injury increased dramatically at 10 dpi (Fig. 2 D,d) and later at 14 dpi these cells were found within the perilesional area (Fig. 2 E,e). Additionally, these results were corroborated by BrdU labeling. The results showed that BrdU^+^/DCX^+^ cells were observed within the dorsal area of the SVZ lining the corpus callosum (Additonal file 1: Fig. S3 A,a). As soon as 5 dpi, DCX^+^/BrdU^+^ cells were observed leaving the dorsal SVZ and crossing the corpus callosum (Additonal file 1: Fig. S3 B,b). At 7 dpi, DCX^+^/BrdU^+^ cells were observed that had crossed the corpus callosum towards the injury (Additonal file 1: Fig. S3 C,c). At 14 dpi a chain of DCX^+^ cells were observed in the region between the corpus callosum and the injury (Additonal file 1: Fig. S3 D,d). Some of these cells retained the BrdU labeling. No DCX^+^ cells were observed in this area at 28 dpi (Additonal file 1: Fig. S3 E). These observations indicated that some of the neuroblasts observed within the perilesional area had migrated from the SVZ.

**Fig. 3:**
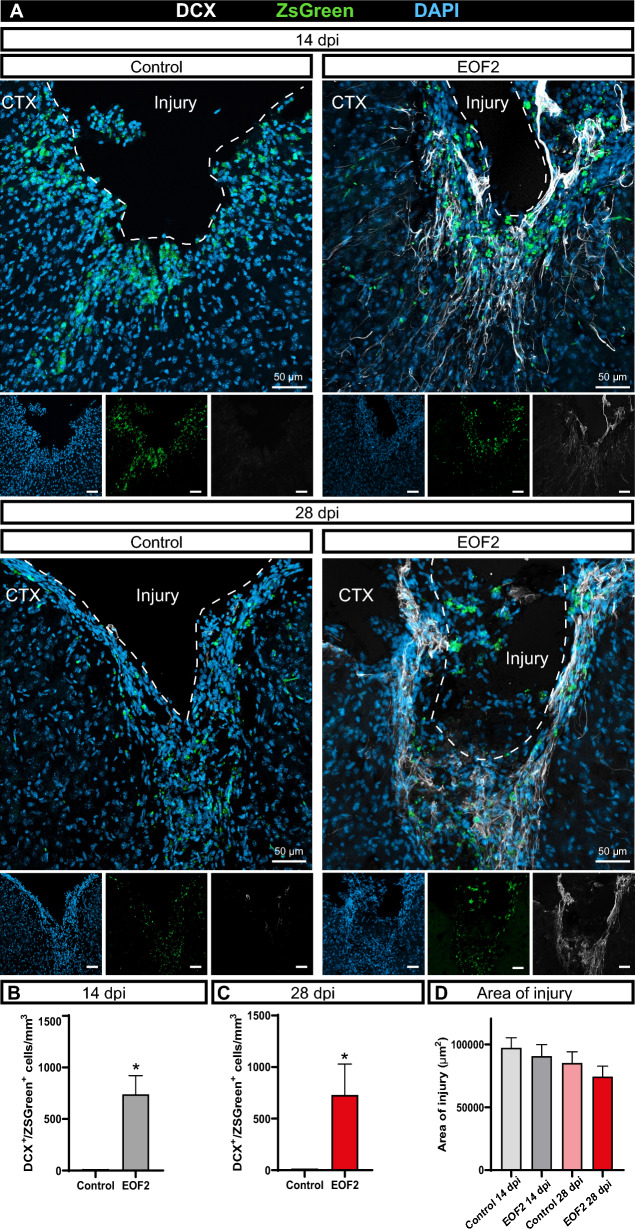
Intranasal EOF2 administration results in an enrichment in the number of neuroblasts within the perilesional area.** A** Representative confocal images of adult mice cortex after bearing mechanical lesions and the intranasal administration of vehicle (control) or EOF2 for 14- or 28-days post injury (dpi). Immunohistochemistry was performed for the detection of the nuclear marker DAPI, the neuroblast marker DCX and ZsGreen. A lentiviral vector expressing ZsGreen was injected in the lateral ventricle ipsilateral to the injury to mark SVZ cells in the same surgical procedure of the injury. Scale bars represent 50 µm. CTX = cortex. **B** Quantification of DCX^+^/ZsGreen^+^ cells/mm^3^ in the perilesional area after 14 dpi in mice treated with vehicle (control) or EOF2. Data show the mean ± SEM of 6 animals per group. Statistical analysis: * p = 0.0024 in Student’s t test for equal-variance unpaired samples. **C** Quantification of DCX^+^/ZsGreen^+^ cells/mm^3^ in the perilesional area after 28 dpi in mice treated with vehicle (control) or EOF2. Data show the mean ± SEM of 6 animals per group. Statistical analysis: * p = 0.0241 in Student’s t test for equal-variance unpaired samples. **D** Quantification of the area of the injury in mice treated with vehicle (control) or EOF2 for 14 or 28 dpi. Non-statistical significances were shown in one-way ANOVA

We next analyzed whether this migration resulted in a larger number of neuroblasts within the injured area after prolonged periods of treatment. We observed that at 14 dpi, the presence of a large number of neuroblasts was found within the perilesional area of EOF2 treated mice. On the contrary, no neuroblasts were observed within the perilesional area in non-treated mice. The number of ZsGreen labeled neuroblasts were analyzed in the injured cortex and the SVZ. As shown in the Fig. 3 (Fig. 3 A,B), at 14 dpi, a set of ZsGreen labeled DCX^+^ neuroblasts was observed within the perilesional area of EOF2 treated mice (right upper panel) that was not observed in control mice (left upper panel). At 28 dpi DCX^+^/ZsGreen^+^ cells were still observed in EOF2 treated mice but not in control mice. As a result, in EOF2 treated mice DCX^+^ labeled with ZsGreen were found at 14 and 28 dpi (Fig. 3 A-C). Furthermore, there is a tendency for the area of the injury to decrease over time, with this decrease being more evident in animals treated with EOF2 than in untreated animals (Fig. 3 D). To test whether the presence of newly generated neuroblasts resulted in an increased number of newly generated neurons within the perilesional area, we analyzed the total number of NeuN^+^ cells that contained ZsGreen labeling (double labeled ZsGreen^+^/NeuN^+^ cells). We observed that in at 28 dpi, the total number of double labeled ZsGreen^+^/NeuN^+^ cells increased by twofold compared to 14 dpi (Additonal file 1: Fig. S4) suggesting it may be a consequence of the differentiation of the neuroblasts observed in Fig. 3.

### Functional properties of newly generated neurons within the perilesional area

**Fig. 4:**
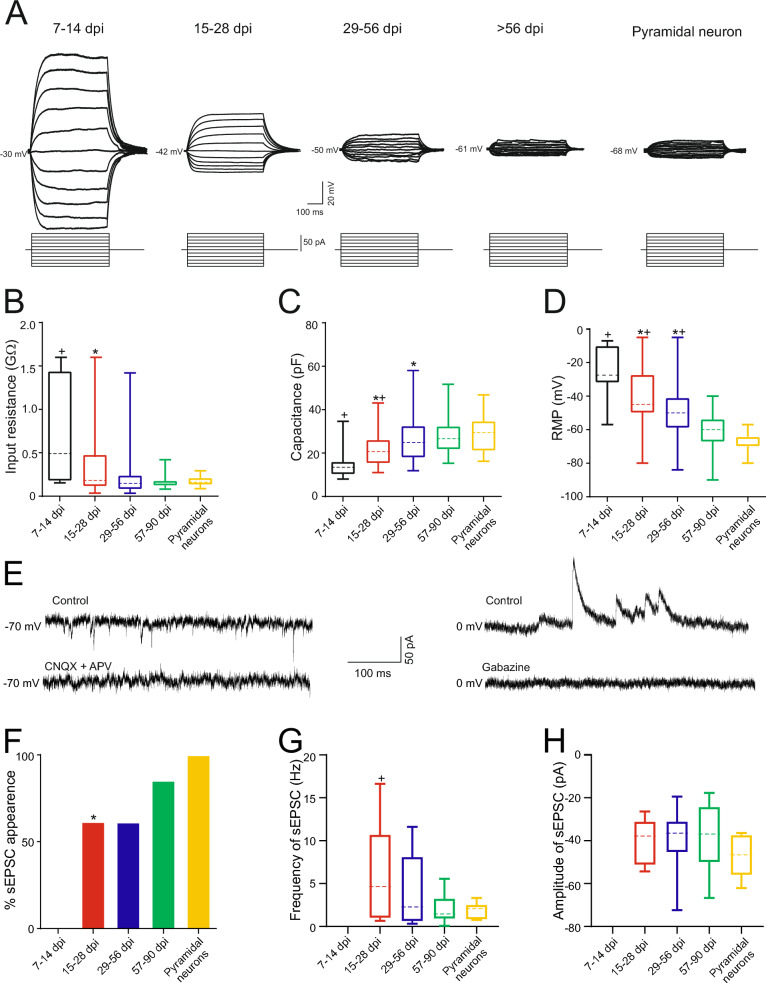
Passive membrane properties of newly generated neurons and integration into local synaptic circuits. **A** Membrane potential response to depolarizing and hyperpolarizing current pulses in a typical cell from each experimental group. See the big difference in the response of the first group compared to the others. **B-D** Box-and-whisker plots showing the medians (dashed lines), interquartile ranges (boxes), minimum/maximum values (whiskers), and SEM (error bars) for input resistance (B), capacitance (C), and resting membrane potential (RMP, D), of each experimental group. Resistance decreased drastically between the 7–14 days post-injury (dpi) group and the 15–28 dpi group (*p = 0.0154). Capacitance increased gradually between the first and second groups (*p = 0.034) and between the second and third groups (*p = 0.04). RMP progressively decreased until 90 dpi, with significant differences observed between the first and second groups (*p = 0.0043), the second and third groups (*p = 0.001), and the third and fourth groups (*p = 0.0003). No differences were found in any parameter between the 57–90 dpi group and the pyramidal neurons group. Statistical analysis: Repeated measures ANOVA with Tukey post-hoc. **E** Image showing the glutamatergic (left) and GABAergic (right) nature of the synaptic inputs to newly generated neurons. **F** Bar chart showing the percentage of spontaneous excitatory postsynaptic currents (sEPSC) occurrence in each experimental group. This graph shows very clearly how the newly generated neurons begin to integrate into the circuit starting at 15 dpi. Fisher's test was used to assess differences in observed frequencies of synaptic appearance. **G-H** Box-and-whisker plots showing the medians (dashed lines), interquartile ranges (boxes), minimum/maximum values (whiskers), and SEM (error bars) for the frequency (G) and amplitude (H) of EPSC of each experimental group. The frequency of sEPSCs progressively decreased until 90 dpi, with significant differences observed between the 7–14 dpi group and the 57–90 dpi group (+ p = 0.0089). Statistical analysis: Repeated measures ANOVA with Tukey post-hoc. For all graph, the asterisk (*) denotes statistically significant differences between consecutive groups, while the cross ( +) indicates statistically significant differences between that group and the 57–90 dpi group. The significance level was established as p ≤ 0.05

**Fig. 5:**
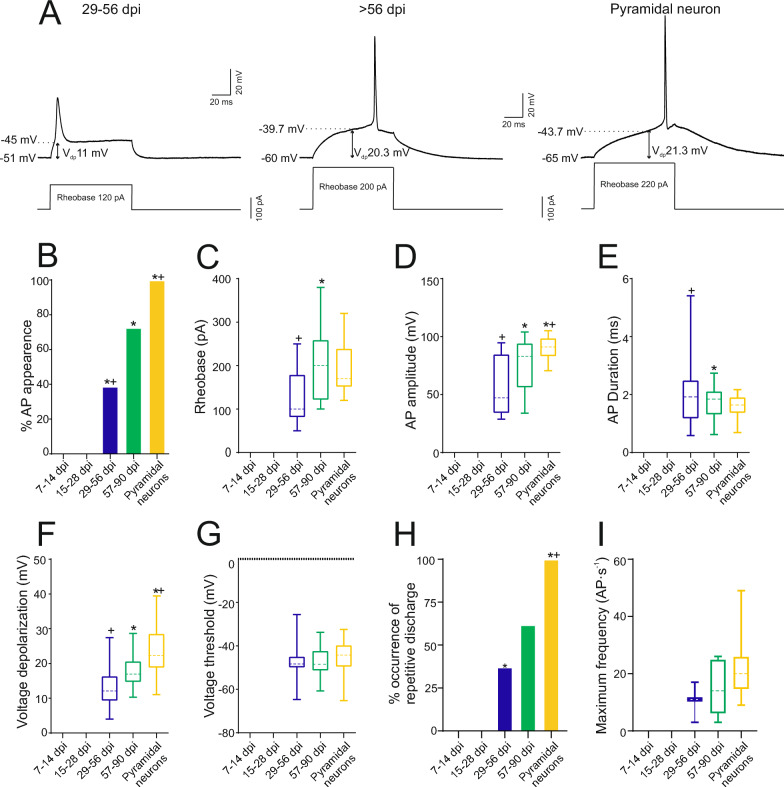
Newly generated neurons become able to fire an action potential starting from 28 dpi and are able to discharge action potentials repetitively from 56 dpi. **A** Membrane voltage responses to the minimum current required to evoke an action potential (rheobase) in a representative neuron from each experimental group. **B** Bar chart showing the percentage of cell firing action potential in each experimental group**.** It can be seen how the cells are able to start firing from 28 days post-injury (dpi)**.** Fisher's test revealed significant differences in the frequency of cells able to fire action potentials between the 15–28 dpi and 29–56 dpi groups (*p < 0.0001), between the 26–56 dpi and 57–90 dpi groups (*p = 0.0074), and between the 57–90 dpi group and pyramidal neurons (*p = 0.0033). **C-G** Box-and-whisker plots showing the medians (dashed lines), interquartile ranges (boxes), minimum/maximum values (whiskers), and SEM (error bars) for rheobase (C), action potential (AP) amplitude (D), AP duration (E), voltage depolarization (F) and voltage threshold (G) of each experimental group. Note that the rheobase increased from the 26–56 dpi group to the 57–90 dpi group (*p = 0.0033), as did the action potential amplitude (*p = 0.006), while the duration decreased (*p = 0.036). Additionally, the action potential amplitude still showed differences between the 57–90 dpi group and the pyramidal neurons group. Statistical analysis: Repeated measures ANOVA with Tukey post-hoc. **H** Bar chart showing the percentage of repetitive response occurrence in each experimental group. We can see how they begin to have repetitive firing properties starting at 29 dpi, although as we see, even above 56 dpi these values are far from those of a pool of pyramidal neurons. Fisher's test was used to determine differences in the observed frequencies of repetitive discharge, revealing significant differences between the 15–28 dpi and 29–56 dpi groups (*p = 0.0045) and between the 57–90 dpi group and the pyramidal neuron group (*p = 0.0039).** I** Box-and-whisker plots showing the maximum frequency values for each group. Statistical analysis: Repeated measures ANOVA with Tukey post-hoc. For all graph, the asterisk (*) denotes statistically significant differences between consecutive groups, while the cross ( +) indicates statistically significant differences between that group and the 57–90 dpi group. The significance level was established as p ≤ 0.05

The analysis specifically focused on the excitability of ZsGreen^+^ cells in the injured motor cortex, examining first their passive membrane properties (Fig. 4 and table S5), followed by their active membrane properties (Fig. 5 and Additonal file 1: Tables S4 and S5) in patch clamp whole-cell recordings. ZsGreen^+^ cells exhibited high values of input resistance (approximately near a 1 GΩ) and low values of capacitance (around 15 pF) up to 14 dpi, indicating their small size at that stage. The input resistance values abruptly decreased by half at 28 dpi and remained at levels typical of control pyramidal neurons from 29 to 56 dpi (Fig. 4 A-B). Similarly, capacitance progressively increased from 15 to 28 dpi and then stabilized at control values from 29 to 56 dpi (Fig. 4 C). Both input resistance and capacitance reflect cell size [31], suggesting that neurons reach their final size by 56 dpi. As input resistance also mirrors the density of ion channels, the sharper decline observed during the 14–28 dpi period may indicate an increment of the density of leakage channels in the neuronal surface, while the increase in size requires more time, extending from 14 to 56 days.

Depolarized resting membrane potentials (approximately −25 mV) were observed at 7 to 14 dpi, and no recorded cells exhibited synaptic inputs, indicating that they had not yet integrated into existing circuits. However, at 28 dpi, less depolarized resting membrane potentials (around −40 mV) were observed, and 50% of the cells recorded at this time point exhibited synaptic bombardment, suggesting they had already integrated into existing circuits (Fig. 4 D, F). The amplitude of synaptic events remained constant over time, while the frequency values of spontaneous excitatory postsynaptic currents (sEPSCs) changed (Fig. 4 G-H). The highest frequency values of sEPSCs were observed in ZsGreen^+^ cells at 28 dpi and progressively decreased to values similar to those found in pyramidal neurons at 57–90 dpi. Beyond 56 dpi, most ZsGreen^+^ neurons received synaptic inputs and exhibited resting membrane potentials around −60 mV, a value characteristic of mature neurons in the motor cortex (Fig. 4 D, F). Synaptic inputs in neurons at 57–90 dpi were identified as either excitatory, as revealed by the complete blockage of inward spontaneous currents using classic glutamatergic inhibitors such as 6-Cyano-7-nitroquinoxaline-2,3-dione (CNQX) and 2-Amino-5-phosphonopentanoic acid (APV) (left panel, Fig. 4 E), or inhibitory, as demonstrated by the abolition of outward spontaneous currents using gabazine (right panel, Fig. 4E).

No active membrane properties were observed in ZsGreen^+^ cells at 7 to 14 dpi, nor in neurons at 28 dpi. However, between 29 and 56 dpi, approximately 40% of recorded cells triggered action potentials (Fig. 5 A, B). When small-amplitude current steps were injected ZsGreen^+^ cells at 29–56 dpi easily generated action potentials. Progressively larger currents were necessary to reach the membrane threshold to elicit action potentials in more mature neurons (later than 56 dpi) due to their higher rheobase values (Fig. 5 A, C). The properties of action potentials also exhibited marked time-dependent maturation. At 29–56 dpi, ZsGreen^+^ cells could only produce single action potentials with immature characteristics (short amplitude and high duration, Fig. 5 A, D, E) in response to prolonged depolarizing current pulses. At this stage, the voltage depolarization was minimal (about 12 mV), but significantly increased to a higher value (about 18 mV), indicating a more hyperpolarized membrane resting potential (Fig. 5 F). Conversely, the voltage threshold remained unchanged near −40 mV across all dpi groups, as it is a characteristic of voltage-dependent sodium channels (Fig. 5 G). Subsequently [57–90 dpi], most ZsGreen^+^ cells exhibited action potentials with duration and amplitude values similar to those found in cortical neurons (Fig. 5 B, D, E), and 58.3% of them displayed repetitive firing properties (Fig. 5 H, I).

**Fig. 6:**
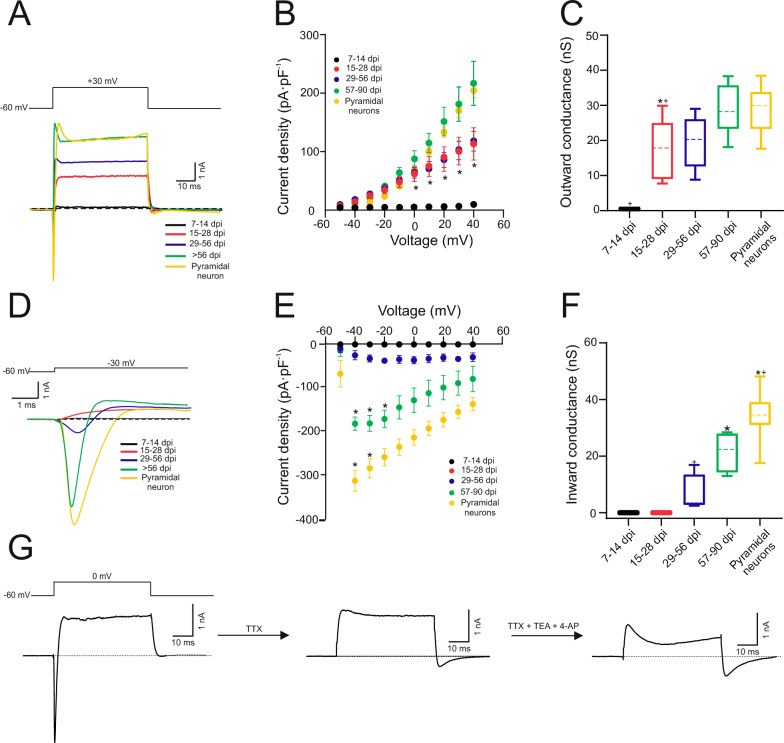
Newly generated neurons display voltage-dependent outward and inward currents. **A**, **D** Representative current responses to + 30 mV (A) and to −30 mV (B) step depolarizations in newly neurons from each experimental group. Holding potential in both cases was −60 mV. **B**, **E** Average current density – voltage relationships for each experimental group for outward (B) and inward currents (E). **C**, **F** Box-and-whisker plots showing the medians (dashed lines), interquartile ranges (boxes), minimum/maximum values (whiskers) and SEM (error bars) for outward (C) and inward (F) chord conductance at + 30 mV and −30 mV, respectively. The most significant difference in outward currents occurs between the first and second groups (*p = 0.034), with further increases and differences between the 15–28 days post-injury (dpi) group and 57–90 dpi group. No differences are observed between the 57–90 dpi group and the pyramidal neuron group. Inward currents appear later in the 29–56 dpi group, increasing in the 57–90 dpi group (*p = 0.0048), but not reaching the values of the pyramidal neurons group (p = 0.018). **G.** Pharmacological dissection of the currents in a representative 57–90 dpi cell. The current was elicited by a step depolarization from −60 to 0 mV. TTX (1 μM), TEA (5 mM) and 4-AP (2 mM) was applied in the external solution. In all cases, statistical differences were analyzed using repeated measures ANOVA and Tukey post-hoc. For all graph, the asterisk (*) denotes statistically significant differences between consecutive groups, while the cross ( +) indicates statistically significant differences between that group and the 57–90 dpi group. The significance level was established as p ≤ 0.05

Continuing the investigation into active properties, we proceeded to examine the voltage-activated currents in the newly generated neurons (Table S5). As depicted in Fig. 6 A, outward currents were practically absent at 7–14 dpi. They began to emerge between 15 and 28 dpi, with their amplitude appearing to increase over the duration of treatment. Both current density and the value of outward chord conductance calculated at + 30 mV exhibited a similar trend, showing an increase over the time of the treatment . However, despite this slight increase in the mentioned parameters after 28 dpi, no statistical differences were found between this group and the 57–90 dpi group or pyramidal neurons (Fig. 6 B, C) suggesting that 28 days cells reach the values of outward density currents typical of mature neurons. On the other hand, Fig. 6 D illustrates that inward currents are only detected in cells older than 29 dpi, statistically increasing their current densities (at −40 and −30 mV voltage pulses) between 29–56 dpi and 57–90 dpi, but not reaching the values of current density found in pyramidal neurons (Fig. 6 E). The value of inward chord conductance calculated at −30 mV increased statistically between 29–56 dpi and 57–90 dpi. This change in inward conductance supports the variation in action potential amplitude, as seen in Fig. 6 D. However, the value of the averaged conductance found at 57–90 dpi was still statistically lower than those found in pyramidal neurons (Fig. 6 F), suggesting that the development of inward current takes a longer time than outward currents to reach control values. To gain a deeper understanding of the nature of the currents, we conducted a classical pharmacological characterization in a small group (n = 5) of 57–90 ZsGreen^+^ cells. As Fig. 6 G illustrates, the application of 1 µM TTX completely abolishes the inward component, indicating that a sodium current was responsible for the inflection of the current. Subsequently, the application of non-specific potassium inhibitors, such as 5mM TEA and 4-Aminopyridine (4-AP, 2 mM), significantly reduces the amplitude of the currents.

### Morphological properties of newly generated neurons in cortical injuries

**Fig. 7:**
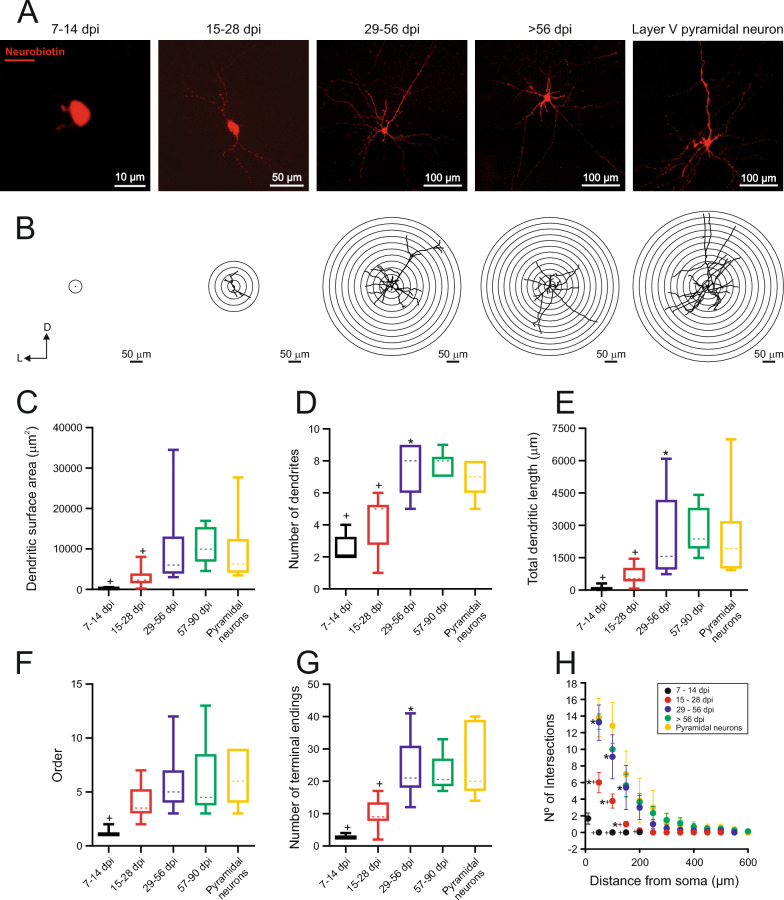
Morphological study of the newly generated neurons. **A** Image of Texas Red anti-neurobiotin stains showing the morphological changes occurring over the course of treatment in newly generated neurons originating from the subventricular zone (SVZ). Note the increase in cell size over the days of treatment, as well as the increase in the number, length and complexity of dendrites. **B** Scholl diagrams showing the morphology of the cells in section A. **C-H** Box-and-whisker plots showing the medians (dashed lines), interquartile ranges (boxes), minimum/maximum values (whiskers) and SEM (error bars) for dendritic surface area (C), number of primary dendrites (D), total dendritic length (E), maximum dendritic branch order (F) and number of terminal endings for each experimental group. Dendritic surface area increased with treatment duration, showing significant differences between the first two groups and the 57–90 days post-injury (dpi) group (+ p = 0.0007; + p = 0.0368). The number of dendrites, their length, and the number of terminals increased over time, with the most significant differences observed between the 15–28 dpi and 29–56 dpi groups (*p < 0.0001, *p = 0.044, and *p = 0.008, respectively). The order increased progressively, with significant differences only when comparing the first group to the 57–90 dpi group (+ p = 0.011). Note how all electrophysiological parameters are established within the first two months of treatment.** H** Dot plot showing the number of intersections versus the distance to soma. Note that in the first and second group these values are very small, but from the third group onwards the values are practically identical to those of a pyramidal neuron. In all cases, statistical differences were analyzed using repeated measures ANOVA and Tukey post-hoc. For all graph, the asterisk (*) denotes statistically significant differences between consecutive groups, while the cross ( +) indicates statistically significant differences between that group and the 57–90 dpi group. The significance level was established as p ≤ 0.05

Besides examining the electrophysiological features of the newly generated neurons, we also aimed to delve into the maturation of their morphological properties (Additional file 1: Table S6). In Fig. 7 A, four representative neuroblasts/new neurons intracellularly labeled with neurobiotin are depicted alongside a layer V pyramidal neuron of the motor cortex. Neuroblasts at 7–14 dpi exhibited round cell bodies with short neurites. New neurons generated at 15–28 dpi displayed two to five primary dendrites emerging from the soma, preferentially oriented in two directions, and could be classified as bipolar neurons. The most frequently observed neurons at 29 dpi and beyond exhibited polygonal cell bodies, with 6 to 9 primary dendrites emerging from various somatic poles, and can be classified as multipolar. The increase in the size of new generated neurons and their dendritic trees is illustrated in Fig. 7 B-E. The aforementioned increase in the number of dendrites produces a significant and pronounced increase in the dendritic surface area (Fig. 7 C). The area undergoes a significant increase from approximately 250 μm^2^ at 7–14 days to 3,000 μm^2^ at 15–28 dpi, followed by an additional significant increase to 10,500 μm^2^ at 29–56 dpi. This value is similar to those observed at 57–90 dpi and those found in pyramidal neurons. Consequently, we can conclude that newly generated neurons reach adult size around 56 dpi.

Dendritic development also resulted in changes in dendritic length, following a temporal pattern similar to the growth in dendritic tree size. Following an initial increase in total length from about 100 µm [7–14 dpi] to approximately 600 µm [15–28 dpi], there was a significant and pronounced increase in length up to about 2,500 microns at 29–56 dpi. These values observed at 29–56 dpi were comparable to those seen in ZsGreen^+^ neurons of the 57–90 dpi group and the control group of pyramidal neurons. Additionally, Fig. 7 H quantified the alterations in dendritic length across dpi using the Sholl diagrams. After 7–14 days, neuroblasts arranged their neurites within a 50 µm radius surrounding the soma. By 15–28 dpi, the maximum dendritic intersections extended to 150 µm, with an average of 6 intersections per neuron. At 29–58 dpi, the maximum dendritic intersections extended to 500 µm, with the peak number of dendritic intersections reaching 13, both values very similar to those observed at 57–90 dpi and in the control group of pyramidal neurons. The arborization pattern of newly generated neurons was also altered with dpi, as evidenced by variations in the number of terminal endings, number of nodes, and branch order, all of which increased to reach values at 29–56 dpi that were substantially similar to those observed later at 57–90 dpi and in the control group of pyramidal neurons (Additional file 1: Table S6). Therefore, the temporal course leading to the increase in size, length, and dendritic complexity was essentially the same. These findings demonstrate that the neuroblasts tended to develop new principal dendrites that elongated and increased in complexity with dpi, resulting in dendritic trees resembling those of mature neurons by 29–56 dpi.

### Relationship between the morphofunctional properties of newly generated neurons and the layer of the motor cortex where they reside

**Fig. 8:**
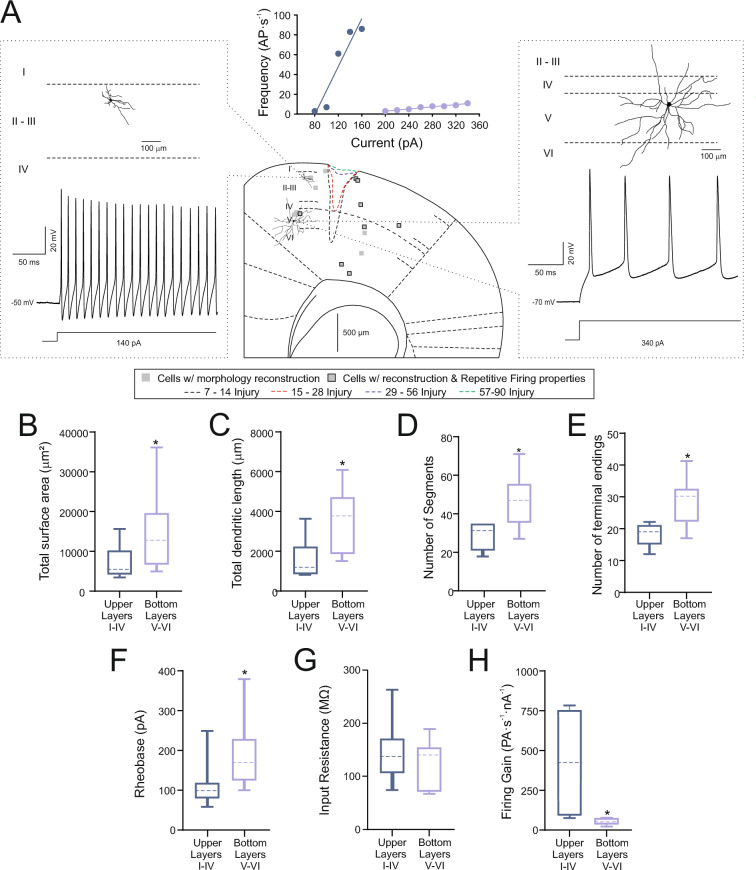
Morphological and electrophysiological distinctions between newly generated neurons in the upper and deeper layers. **A** The figure illustrates the morphological and electrophysiological differences based on neuron location. Neurons in the upper layers (left) are smaller than those in the lower layers (right). Lower layer neurons exhibit more, longer, and more complex dendrites. Additionally, their firing patterns differ, with upper layer neurons showing higher frequency firing, as indicated by the frequency recordings and frequency-current graph at the top. The central panel shows the distribution of recorded neurons from the 29–56 days post injury (dpi) and 57–90 dpi groups. Dashed lines illustrate lesion size changes over time: initially larger at 7–14 dpi (black), and progressively smaller with treatment [15–28 dpi in red, 29–56 dpi in blue, 57–90 dpi in green]. **B-H** Box-and-whisker plots displaying the medians (dashed lines), interquartile ranges (boxes), minimum/maximum values (whiskers) and SEM (error bars) for total surface area (B), dendrite length (C), number of total segments (D), number of terminal endings (E), rheobase (F), input resistance (G), and firing gain (H) for each group. It is remarkable that neurons in deeper layers were larger (*p = 0.036), longer (*p = 0.013), and more complex (segments, *p = 0.004; endings, *p = 0.034). They had higher rheobases (*p = 0.043) and lower firing frequencies (*p = 0.0034). Student's t-test assessed differences between groups. Asterisks (*) indicate statistically significant differences between groups, with a significance level of p ≤ 0.05

Using the methodology described in Additonal file 1: Fig. S1 we closely inspected the morphological characteristics of the newly generated neurons. As illustrated in Fig. 8, the results indicated that the dendritic spatial distribution depended on their distribution in the cortex. The Fig. 8 A illustrates 2 typical neurons localized in layer 2 and a neuron within the landmark of layer V. When we segregated the new ZsGreen^+^ neurons into two groups, those that were installed in the four most superficial layers (I-IV), and those that were installed in the two deepest layers (V-VI) of the cortex, great differences were observed in their morphofunctional characteristics. The new neurons in layer V-VI presented a significantly greater total surface area, total dendritic length, number of total dendritic segments and number of terminal endings (Fig. 8 B-E and Additonal file 1: Table S7) than the new neurons that are installed in the most superficial layers. It is also striking that the most superficial neurons have smaller values of rheobase and a higher values of input resistance and mean firing frequency of action potentials (rheobase 90 vs 165 pA, input resistance 163 vs 121 MΩ and mean firing frequency 433 vs 55 AP/nA) than those ZsGreen^+^ neurons that are installed in the deeper layers. This data would support the conclusion that new generated neurons developed a different phenotype depending on the layer where they are included.

### Expression and release of neuregulin within the perilesional area is stimulated by EOF2

**Fig. 9:**
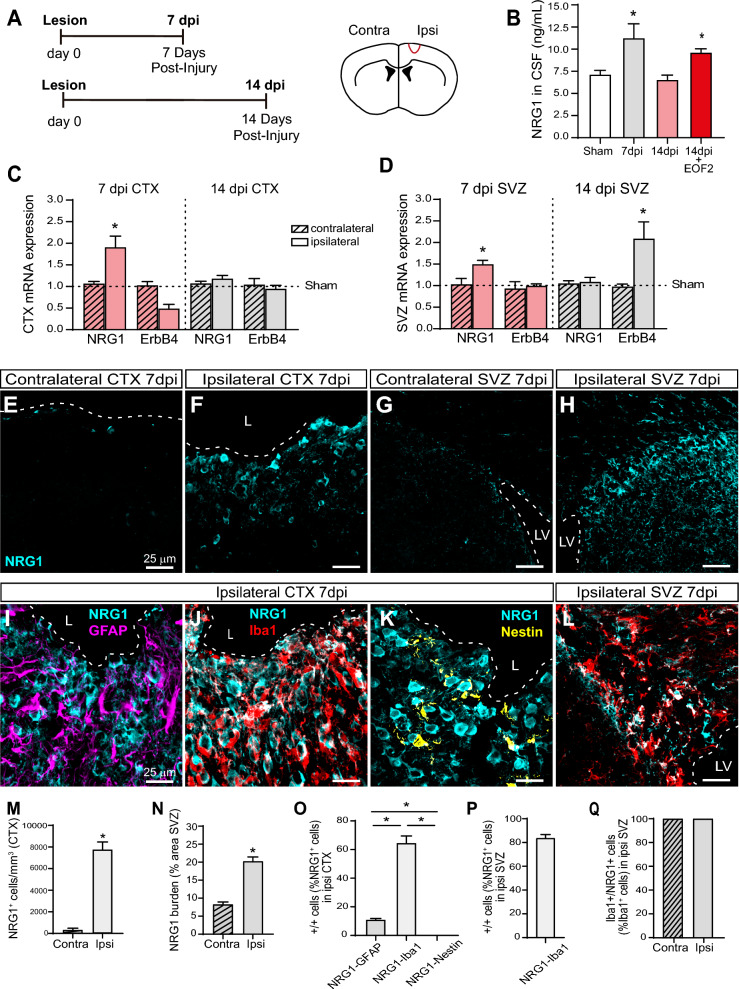
Elevated NRG1 expression in the SVZ and cortex of injured mice, localized in microglial cells and detected in the CSF. **A** Scheme of experimental procedures. Mechanical cortical lesions were unilaterally performed in the adult mouse primary motor cortex (CTX). Mice were sacrificed 7- or 14-days post injury (dpi). Only the group “14 dpi + EOF2” was intranasally treated with EOF2 5 µM from the day of the injury until sacrifice. **B** Mouse cerebrospinal fluid (CSF) were obtained from non-injure mice (sham) and from injured mice at day 7 and day 14 post-injury (dpi). The graph shows neuregulin 1 (NRG1) detection (ng/mL) in CSF. *p = 0.04 Sham vs. 7dpi; p = 0.042 Sham vs. 14dpi + EOF2 in one-way ANOVA**.** 6 animals were used per group. **C** Relative mRNA expression of NRG1, and ErbB4 in the injured cortex (ipsilateral, ipsi) and in non-injured cortex (contralateral, contra) at 7 dpi and at 14 dpi. mRNA expression was measured using real time qPCR. *p = 0.042 7dpi CTX NRG1 (contra vs. ipsi). 6 animals were used per group **D** Relative mRNA expression of NRG1 and ErbB4 in the subventricular zone (SVZ) of the injured ipsilateral side and non-injured side 7 dpi and a 14 dpi. mRNA expression was measured using real time qPCR. The data show the means ± SEM of 6 animals per group. *p = 0.0472 7dpi SVZ NRG1 contra vs. ipsi; *p = 0.0313 14dpi SVZ ErbB4 contra vs. ipsi. **E–H** Representative confocal images showing the CTX (E, F) and SVZ (G, H) of 7 dpi mice. Images were processed for the immunodetection of NRG1. The dotted line indicates the limit of the lesion (L) or lateral ventricle (LV), and the scale bar represents 25 μm. **I-L** Representative confocal images showing the colocalization of NRG1 with GFAP (I), Iba1 (J,L) and Nestin (K) in the Cortex CTX (I-K) and SVZ (L) of 7 dpi mice. Scale bars represent 25 µm. **M** Graph represents the number of NRG1 cells in the contralateral cortex compared to the perilesional area of 7 dpi mice. The data show means ± SEM of 6 animals per group. Statistical analysis: *p < 0.0001 compared the ipsilateral site with the contralateral side in a Student’s t test for equal-variance unpaired samples. **N** Graph shows NRG1 burden as a percentage of the total SVZ area in contralateral and ipsilateral SVZ of 7 dpi mice. Data show Means ± SEM of 6 animals per group. Statistical analysis: *p < 0.0001 compared the ipsilateral site with the contralateral side in a Student’s t test for equal-variance unpaired samples. **O** Quantification of the colocalization of NRG1 with GFAP, Iba1 or Nestin in the injured cortex of 7 dpi mice. The data show the means ± SEM of 6 animals per group; *p < 0.0001 ipsi NRG1^+^GFAP^+^ vs NRG1^+^Iba1^+^; *p = 0.0431 NRG1^+^GFAP^+^ vs NRG1^+^Nestin^+^; *p < 0.0001 NRG1^+^Iba1^+^ vs NRG1^+^Nestin^+^ in one-way ANOVA, followed by posthoc Tukey test. **P** Quantification of the colocalization of NRG1 with Iba1 in the ipsilateral SVZ of 7 dpi mice. Data are the mean ± SEM; n = 6 animals per group. **Q** Percentage of the number of Iba1^+^ cells per mm^3^ that also express NRG1 (Iba1^+^NRG1^+^ cells per mm^3^) in contralateral and ipsilateral SVZ of 7 dpi mice. n = 6 animals per group

As a final attempt, we aimed at elucidating the mechanism by which EOF2 exerts its effect. EOF2 is a diterpene with the capacity to activate novel PKC. The activation of novel PKC leads to the release of neuregulins and competes with the release of other growth factors such as TGFα or HB-EGF that are released in response to classical PKC activation [40, 41]. Since NRG1 exerts an effect as a cell attractant [42], we started by analyzing the expression of NRG1 and its receptor the ErbB4 receptor in the SVZ and within the injured cortex at 7 and 14 dpi. As shown in Fig. 9, a two-fold increase in the expression of NRG1 was found within the ipsilateral injured cortex compared to the contralateral or the sham-operated mice (Fig. 9 A, C). The expression levels of NRG1 in the injured cortex had returned to basal levels 14 dpi (Fig. 9 A, C). No increase in the ErbB4 receptor was observed at 7 dpi (Fig. 9 A, C), on the contrary a non-statistically significant reduction on ErbB4 expression was found at 7 dpi that was not observed at 14 dpi (Fig. 9 A, C). Regarding the SVZ, NRG1 expression increased by 1.5-fold at 7 dpi returning to basal levels at 14 dpi. Interestingly, ErbB4 expression in the SVZ was not altered at 7 dpi, however it had increased by twofold at 14 dpi (Fig. 9 A, D). Immunohistochemistry studies show that the number of cells that expressed NRG1 in the ipsilateral cortex increased by eightfold compared to the contralateral at 7 dpi (Fig. 9 E, F, M) Likewise, the area occupied by NRG1 staining increased by threefold in the SVZ at 7 dpi (Fig. 9 G, H, N). Interestingly, the study of the phenotype of NRG1^+^ cells showed that at 7 dpi, more than 60% of NRG1^+^ cells were Iba1^+^, whereas only 20% were GFAP^+^ and none of them were nestin^+^ (Fig. 9 I-K, O). Equally, more than 80% of NRG1^+^ cells in the SVZ were Iba1^+^ cells (Fig. 9 L, P) and interestingly 100% of Iba1 cells expressed NRG1. Thus, our results suggested that the injury facilitated the appearance of Iba1^+^ microglial cells at the SVZ and the perilesional area that expressed NRG1. We next elucidated whether the elevated expression of NRG1 led to an increased concentration of NRG1 in the CSF. We observed that at 7 dpi NRG1 concentration increased by twofold in the injured mice compared to control mice (sham) (Fig. 9 B). This concentration returned to basal levels at 14 dpi (Fig. 9 B). Interestingly, the treatment of mice with EOF2 was able to maintain NRG1 concentration elevated at 14 dpi (Fig. 9 B). Finally, since EOF2 facilitates the release of NRG1 through activating PKC delta (PKCδ) [43], we tested total (pan-PKC) activity in the ipsilateral and contralateral cortex and SVZ, we found that total PKC activity was elevated in the ipsilateral cortex compared to the contralateral at both 7 and 14 dpi (Additonal file 1: Fig. S5 A). Accordingly, the expression of PKCδ increased in the ipsilateral cortex of injured mice at both 7 and 14 dpi (Additonal file 1: Fig. S5 C). Notwithstanding, PKC activity was elevated in the ipsilateral SVZ at 7 dpi but not at 14 dpi whereas PKCδ expression was only elevated in the ipsilateral SVZ at 14 dpi (Additonal file 1: Fig. S5 B).

### In vitro release of neuregulin from SVZ-isolated cells is stimulated by EOF2

**Fig. 10:**
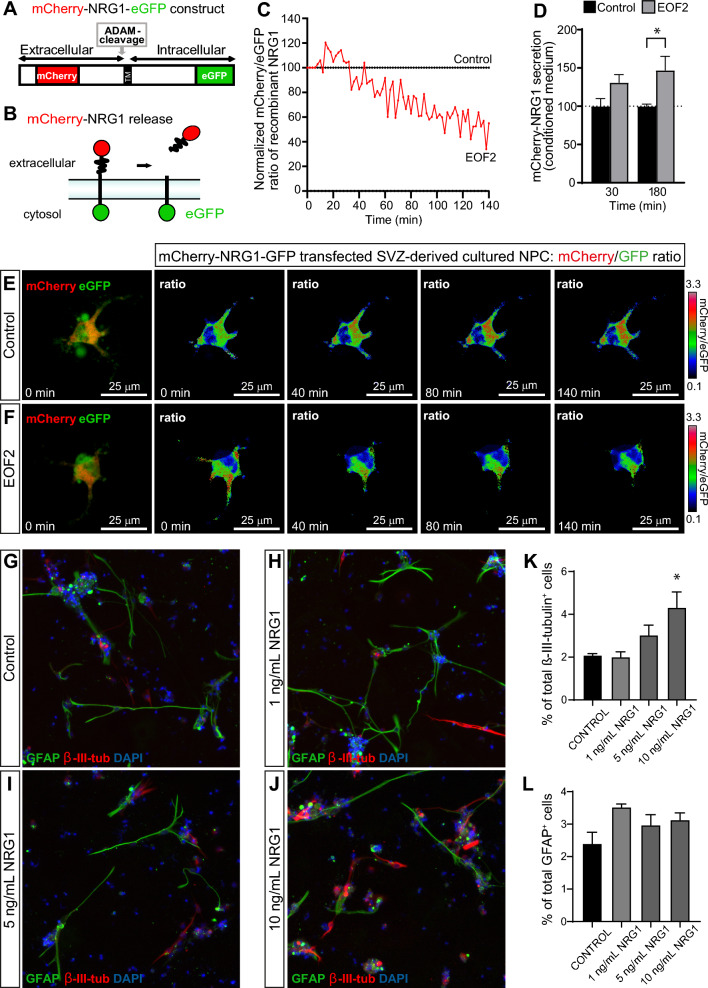
Molecular mechanisms underlying the effects of EOF2: role of NRG1. **A** Scheme of mCherry-NRG1-eGFP construct. **B** Mechanisms of NRG1-bound fluorescence release. **C** Quantitative analysis of the microscopic images obtained from the time-lapse assays of neural progenitor cells (NPC) expressing mCherry-NRG1-eGFP and stimulated with EOF2 (5 μM). mCherry/eGFP ratios were normalized to the average mCherry/eGFP ratio measured before stimulation. The mean normalized mCherry/eGFP ratios are shown, n = 3. At least 10 cells were analyzed per condition in each experiment. D. mCherry fluorescence in the culture medium of cells transfected with the construct mCherry/eGFP treated with EOF2 for 30 and 180 min. **E–F** mCherry/eGFP ratio images of SVZ-derived cultured NPC treated with diluent (control) or EOF2 (5 µM) are shown in the intensity-modulated display mode at the indicated time points. The color range goes from red to blue to represent mCherry/eGFP ratio. The upper and lower limits of the ratio range are shown. Scale bar represents 25 μm. **G-J** Representative fluorescence images of SVZ-derived cultured NPC treated with diluent (control) or the soluble NRG1 ligand (1, 5 or 10 ng/mL). Cells were grown without growth factors and allowed to differentiate for 72 h after treatment. β-III-tubulin marker was used for neuronal immunodetection (red), and glial cells were identified by the immunodetection of GFAP (green). Total nuclei were counterstained with DAPI (blue). **K** Graph represents the percentage of total cells (detected by DAPI nuclear staining) that were positive for β-III-tubulin. **L** Graph represents the percentage of total cells (detected by DAPI nuclear staining) that were positive for GFAP. Data are the mean ± SEM of six independent cultures (n = 6). Differences were detected by one-way ANOVA followed by the Tukey b test vs control (*p = 0.0063). All in vitro experiments were done in triplicate. A triplicate was considered a unit and six triplicates were used for the statistical analysis

These results suggested that EOF2 was responsible for inducing NRG1 release in these cells. In order to demonstrate the effect of EOF2 on NRG1 release we isolated cells from the SVZ that were cultured as neurospheres for three consecutive passages and cultured attached onto a substrate. These cells were then transfected with an expression vector that contained the sequence of a fusion protein in which NRG1 was expressed flanked by eGFP and mCherry in the C-terminal and N-terminal domains respectively (Fig. 10 A). The expression of this construct results in the integration in the plasma membrane of a NRG1 protein in which the C-terminal and N-terminal domains a fluorescent and the ratio mCherry/eGFP is 1 (Fig. 10 B). Addition of EOF2 to the culture medium results in a reduction of the mCherry/eGFP ratio due to the induced release of NRG1 and the consequent loss of mCherry fluorescence (Fig. 10 C, E and Additonal file 2: Movie S1). No reduction in mCherry/eGFP ratio was observed in control cultures (Fig. 10 C, D and Additonal file 3: Movie S2). To ensure that EOF2 was stimulating the release of NRG1, we used Hek293 cells transfected with the mCherry/eGFP construct. These cultures were incubated with EOF2 for 30 and 180 min and mCherry fluorescence was detected in the culture medium afterwards. An increase in mCherry fluorescence was found in the culture medium after 180 min of incubation (Fig. 10 D) supporting that EOF2 stimulates NRG1 release.

### Neuregulin-induced differentiation of SVZ isolated cells

These results indicated that EOF2 facilitated NRG1 release in SVZ derived cells, thus, we analyzed whether NRG1 induced differentiation in these cells. Cells isolated from the SVZ and cultured as neurospheres were grown attached onto a polyornithine substrate for 72 h in the absence of growth factors and in the presence and absence (control) of NRG1. We found that the percentage of beta-III-tubulin^+^ neuroblasts in control cultures was around 2% (Fig. 10 G, K) whereas the treatment with increasing concentrations of NRG1 increased this percentage by two-fold (Fig. 10 H–K). On the contrary, the percentage of GFAP^+^ cells did not increase with the treatment (Fig. 10 G-J, L). These results indicated that NRG1 facilitated differentiation of SVZ isolated cells in agreement with the in vivo findings. Notwithstanding, the quantification of the proportion of Iba1^+^ cells that expressed NRG1 in the SVZ revealed that all Iba1^+^ cells express NRG1, indicating that the arrival of microglial cells at the injured SVZ will result in an increased expression of NRG1 that could be released to the extracellular medium.

## Discussion

In the present work, we have used a viral labeling technique that allows reliable identification of newly generated neurons in cortical injuries. We have found neuroblasts that migrate from the SVZ towards the motor cortex in animals treated with EOF2, which differentiate into fully mature neurons in a time-dependent manner that recapitulates embrionic/neonatal development. These cells receive functional afferents, discharge action potentials in response to a depolarizing excitation, and develop an adult phenotype that corresponds to the layer they are installed on. This complete set of functional properties endows newly generated neurons with the capacity to play a significant role in repairing cortical lesions. As an attempt to elucidate the mechanisms involved in the EOF2-induced differentiation we have deepen into the role that microglial cells play in the release of NRG1 and the capacity of EOF2 to stimulate the release this chemoattractant.

### Injury-induced SVZ neurogenesis is enhanced by EOF2 treatment

A large number of previous evidences show that cortical injuries stimulate neurogenesis in the SVZ [1, 6, 9, 10, 14, 44]. The results shown here, with the use of lentiviral vectors support these findings. We have observed that in response to the cortical injury performed a larger abundance of neuroblasts (DCX^+^) that incorporated ZsGreen was observed in the SVZ demonstrating that cortical injuries stimulate neurogenesis in this region. In agreement with previous findings these neuroblasts were found in the OB, 14 and 28 days later [45] but no DCX^+^ cells were observed in control mice by the injured region at these timepoints. Interestingly, the treatment with EOF2 significantly incremented SVZ neurogenesis, particularly in the ipsilateral SVZ generating neuroblasts that migrate not only to the OB but also to the injured area.

### The migration pattern of neuroblasts is altered in response to the injury and EOF2 treatment

The SVZ is an established rich source of new olfactory neurons [46]. Physiologically, NSC in the SVZ provides neuroblasts that migrate through the RMS towards the OB where they differentiate into mature olfactory interneurons. Notwithstanding, under pathological conditions, such as ischemic or traumatic injuries, neuroblasts of the SVZ may alter their migration pattern migrating toward the injured area to provide newly generated neurons. Previous evidence show that in response to local ischemia the SVZ generates neuroblast that migrate to the striatum where they differentiate into mature neurons following the infusion of growth factors in the lateral ventricles [16]. Moreover, the SVZ has been shown to provide neurons to cortical injuries upon the modulation of signaling cascades that depend on ADAM17 [14]. We show here that the SVZ responds to an injury by increasing the number of newly generated neuroblasts and that this response is enlarged when stimulated by diterpene EOF2. Our results also show that the number of newly generated SVZ neuroblasts is reduced from 3 dpi compared to 14 dpi indicating that these cells are migrating either towards the OB or towards the perilesional area. In agreement, we show in Additonal file 1: Fig. S2 that these cells migrate to the OB and ZsGreen labeled neuroblasts are found at 14 and 28 dpi, being higher in the ipsilateral OB than in the contralateral and in EOF2 treated mice compared to control. Thus, an effect of the injury on neuroblast migration towards the OB was observed that was increased by EOF2 treatment.

Likewise, we also found newly generated neuroblasts within the injured area, which appear to be migrating from the SVZ towards the injury in a time dependent manner providing the injured motor cortex with newly generated neuroblasts as soon as 10–14 dpi [9]. Neuroblasts seemed to be leaving the SVZ and crossing the corpus callosum around day 5 post injury. Many appeared to be migrating to the damaged motor cortex on day 7 post injury reaching the lesion by day 14 following the treatment with EOF2. On day 14 the perilesional area appears replenished of neuroblasts that by day 28 post injury start the differentiation to mature neurons. No migration was observed in the absence of the treatment. Despite the number of neuroblasts that we observed migrating towards the injury, we cannot discard the possibility that some of the neuroblasts found within the perilesional area have generated on site from NSC or progenitors that have migrated to the injured region and have differentiated once they reached the cortex or from activated astrocytes that have been reprogrammed as a consequence of the treatment as observed earlier [47, 48] with brain derived neurotrophic factor (BDNF).

### Neuregulin as a chemoattractant for SVZ neuroblasts

In light of the above-mentioned findings the question arises as to whether the migration of neuroblasts is driven by cell-autonomous mechanisms or mediated by a chemoattractant. Knowledge on the mechanisms that lead neuroblast migration has been obtained by studying the migration of these cells toward the OB. Neuroblasts migrate in chains after acquiring an elongated morphology with a long leading process [49, 50]. According earlier reports SVZ neuroblasts are sessile and need to initiate differentiation in order to migrate long distances [51]. The mechanism of differentiation may be mediated by the receptor of the ErbB family ErbB4, and its ligands neuregulins [42, 52]. NRG2 leads neuroblasts toward the OB whereas NRG1 acts as a chemoattractant leading neuroblasts toward the NRG1 source [42]. We have found here that in response to an injury, microglial cells arrived at the perilesional area and the SVZ. As shown in Fig. 9, microglial cells expressed NRG1. As a consequence, NRG1 expression increased in the SVZ and cortex as soon as 7 dpi, which was accompanied by elevated concentration of soluble NRG1 in the CSF. Both NRG1 expression and the soluble ligand returned to normal levels at 14 dpi. Interestingly, the expression of NRG1 receptor ErbB4 did not increase until 14 dpi, when NRG1 concentration had returned to normal levels. However, the treatment with EOF2 maintained NRG1 concentration elevated in the CSF at 14 dpi. Previous evidence shows that EOF2 induced activation of novel PKCδ promotes NRG1 release via the phosphorylation of the NRG1 pro-ligand [43]. We show in here that protein kinase C activity is increased in the injured SVZ and cortex up to 14 dpi. This is concomitant with an increased expression of PKCδ. These results support the hypothesis that NRG1 release is stimulated by EOF2 through the activation of PKCδ. All these results agree with these previous reports [43]. As the above evidence suggested, there is an increase in the levels of NRG1 in treated mice, not only because of the elevated number of microglial cells that express NRG1 but also by the novel PKC-induced NRG1 shedding driven by EOF2 treatment. Regarding the cellular mechanisms involved in the migration of neuroblasts. Since we observe that they migrate in chains towards the injury, it is possible that they migrate assisted by blood vessels and reactive astrocytes as previously reported in injuries like the ones tested by us [5, 53].

### Newly generated neurons acquire physiological characteristics of matures neurons in a time dependent manner that obeyed embryonic/neonatal development

Our voltage-clamp recordings revealed that newly generated neurons receive excitatory synaptic inputs when they had just arrived to the cortical lesion, indicating that the network formation was established very early and before reaching maturation of the active membrane properties, as also seen in newly generated neurons in dentate granule cells of the hippocampus [54–59] and ischemia in the striatum [16]. The highest frequency values ​​of sEPSCs were found in our study in ZsGreen^+^ newly generated neurons at 28 dpi and decreased progressively to reach values at 57–90 dpi ​​similar to those found in pyramidal neurons. These data seem to indicate that migrating ZsGreen^+^ cells receive input from numerous neurons when they arrive at the motor cortex and a competitive selection may be occurring to define which circuit they will finally integrate into. An alternative explanation could be that the frequency of sEPSCs is the sum of glutamatergic and GABAergic events at 15–28 dpi. It is known that GABA initially depolarizes newborn dentate granule cells in the adult brain, a depolarization that is essential for the establishment of functional synapses [60]. Therefore, we can propose instead that the frequency of sEPSCs would decrease with time as GABAergic currents become inhibitory during the maturation.

Electrophysiological properties of the newly born neurons underwent changes with a similar time course of the maturation of the functional properties as described in developing pyramidal neurons of the motor cortex [61]. The newly generated neurons that we found in the injured motor cortex exhibited immature characteristics, such as a depolarized resting membrane potential, higher input resistance, and are not able to fire action potentials up to 29 dpi. At a later stage, they gradually exhibited significantly more hyperpolarized resting potential, lower values of input resistance and multiple action potentials with depolarizing pulses. This drift in the resting membrane potential could be explained by a higher expression of the K^+^/Cl^−^ cotransporter in mature neurons that would turn GABAergic synaptic inputs from depolarizing to hyperpolarizing [62], [63].

The reduction in input resistance could be due to an enlarged expression of the potassium leak channels and a rise in neuronal size [61, 64]. Outward currents were present in the early stages of maturation in newly generated neurons as reported previously in cultures [65], preceding the appearance of inward currents. The refinement in action potential shape observed in newly generated neurons could be mainly given by an increase in density of the voltage-gated potassium and sodium. This increase in current densities over time resembles the described changes in rat neocortex development [66, 67]. To better understand the nature of the currents, we performed a classical pharmacological characterization. The application of TTX 1 μM abolished completely the inward component, indicating that a sodium current was responsible for the inflection of the current. Although we cannot discard the idea that there were an underlying calcium component, due to the essential role of calcium currents in early stages of the development [68] and the presence in layer V pyramidal neurons [69]. The candidate channels responsible for these inward currents could be Na_V_1.2 [70] or Na_V_1.6 [71, 72], which has been detected in cortical neurons. Following this, the application of non-specific potassium inhibitors, such as TEA (5 mM) and 4-AP (2 mM), significantly reduced the amplitude of the currents. The non-blocked current is probably part of the leakage currents, which are insensitive to these drugs, although it was proposed that there are components of potassium currents partially insensitive to TEA or 4-AP in cortical neurons [73, 74]. While in the adult neurogenic regions (subgranular zone of the hippocampus and SVZ-OB) new neurons acquire mature electrophysiological properties within 4 weeks [54, 75, 76], the newly generated neurons that were found in the dorsolateral striatum after ischemia [16] and in our study in the injured motor cortex displayed repetitive discharge of action potentials within 12–20 weeks. Therefore, the time scale of functional maturation in injured brains was longer than in the adult neurogenic regions. This difference might imply that the local environment after the brain insult was less favorable for new neurons due to the diminution of trophic support. Therefore, this hostile environment of the traumatic brain injury itself can cause effects in the expression [77] and characteristics of the biophysical properties of voltage-gated ion channels [78].

### Newly generated neurons develop morphological characteristics similar to those of mature neurons in a time-dependent manner, resembling the processes observed during embryonic and neonatal development

The available data on the development of dendritic trees in newly formed neurons in adult brains are limited, mainly coming from the adult dentate gyrus neurogenic niche [79, 80]. Neuronal differentiation in the dentate gyrus follows a pattern similar to that described in embryonic stages. New neurons acquire their final morphological characteristics over several months, going through stages of migration, dendrite and axon formation, dendritic and axonal growth, synapse establishment, and synaptic modifications [59, 76]. In the piriform cortex, immature (dormant) neurons mature with an increase in length and complexity of the dendritic tree lasting for 6 months [81, 82]. During postnatal development, neurons in newborn animals experiment morphological changes as they mature throughout this period [29, 34, 83]. Postnatally, the dendritic arbor of both hypoglossal and oculomotor motoneurons grows in size and length towards areas from which new synaptic inputs originate, but the complexity of their dendritic arbor is already established at birth [29, 34] as also described in hippocampal interneurons [84] or cortical neurons in the visual or prefrontal cortex [85, 86]. By contrast, in our study migrating new generated neurons experience an increase in dendritic complexity. Cells resemble the typical morphology of neuroblasts with rounded soma and very short dendritic processes when arrived to the motor cortex, and then became bipolar and finally reach a dendritic arborization with a dendritic complexity that resembled mature cortical neurons, at a time when they do not yet display fully inward currents. We hypothesize that the augmentation in dendritic complexity may mirror a phenomenon that takes place during embryonic cortical development [87]. Subsequently, the gradual enlargement and elongation of dendrites in these newly generated neurons could be linkened to the processes found during postnatal development [29].

### Neurons developed the morphofunctional characteristics of the cortical layer in which they are integrated

In the cerebral cortex of adult animals, neurons are organized into six distinct layers with differences in morphology, size and cell density [88, 89]. In our study we found that neurons located in the deeper layers (V-VI) exhibited larger and higher rheobase and lower input resistance compared to neurons in the upper layers (I-IV). These results support the idea that cells in layers V-VI must have a larger size and less excitability than those in layers I-IV. The increase in rheobase and a decrease in input resistance during postnatal development is related to an increase in size [61, 86, 90]. Therefore, newly generated neurons installed in the upper layers undergoes a differential pattern of development compared to those located in the deeper layers. The firing pattern of neurons in layers I-IV was also characterized by high instantaneous frequency and frequency gain, similar to the pattern observed in cortical interneurons, highly excitable cells [91]. On the other hand, newly generated neurons located in the lower layers showed a lower number of action potentials per unit of time, similar to the pattern observed in pyramidal neurons [61, 92]. Based on their morphofunctional properties, it could be suggested that newly generated neurons in the upper layers may have differentiated mainly into interneurons, while those in the lower layers may have differentiated mainly into pyramidal neurons. Our results, that neurons develop phenotype of the region in which they are embedded, are consistent with those obtained in mice subjected to ischemia in the striatum [16] when they were treated with growth factors. The integration of these new neurons into the ischemic area resulted in an improvement in motor function in rodents, attributed to the regeneration and repair of the striatum. Similarly, following a stroke in the motor cortex, local manipulation of cellular activity with growth factors and physical training in the affected limb promoted neuronal migration, differentiation, and maturation, preceding functional recovery post-stroke [93]. Our findings and data from other studies suggest that the nature of the inputs may regulate the maturation of migrating cells in a activity-dependent and areal-specific manner [94], which strongly contrast with those that suggest the phenotype is intrinsically determined prior to settling within the cortex and therefore the afferents they receive are dictated by cell type [95, 96].

## Conclusion

These results significantly contribute to understanding the effect diterpenoid EOF2 in cortical injury repair. This small molecule facilitates the release of NRG1, expressed by microglial cells in the perilesional area and promotes the migration of neuroblasts from the subventricular zone towards the lesion site and their subsequent differentiation. The findings obtained in our study show that neurons generated in the SVZ and migrating towards the injury undergo a gradual morphological evolution, from immature neuroblasts to mature neurons with characteristics similar to cortical cells. Additionally, there is evidence of a correlation between the morphological and electrophysiological properties of these neurons, suggesting specific adaptations depending on the layer of the cerebral cortex in which they are located. Thus, our results highlight the role of diterpenoids as a potential therapy to repair cortical injuries.

## Supplementary Information

Additional file 1.

Additional file 2.

Additional file 3.

## Data Availability

Data for each figure have been filed and will be available upon request.
