# Supplementary material for: The subventricular zone neurogenic niche provides adult born functional neurons to repair cortical brain injuries in response to diterpenoid therapy
Source: Stem Cell Res Ther. 2025 Jan 5;16:1. doi: 10.1186/s13287-024-04105-4 (PMC11702051; doi:10.1186/s13287-024-04105-4)
Supplement: Supplementary file 1 — Additional file 1. [file 13287_2024_4105_MOESM1_ESM.pdf]

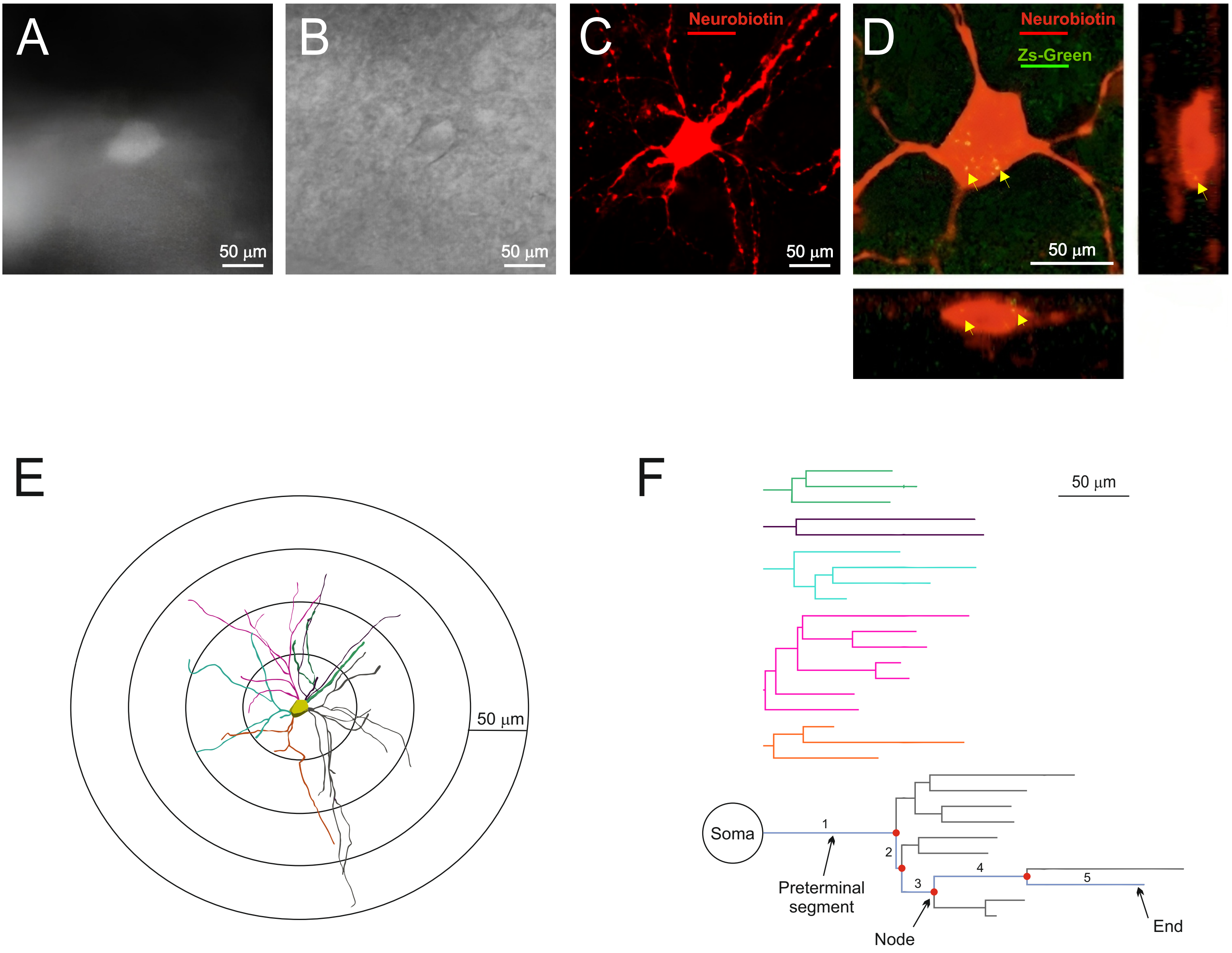

Supplementary Figure S1. Methodological approach for morphological analysis of newly generated neurons. A-D. Images showing the same cell labelled with ZsGreen observed by fluorescence (A), infrared microscopy (B), neurobiotin staining (C), and Neurobiotin (red) and ZsGreen (Green) staining (D). In D we can also see the ortho projections of the cell. The yellow arrows highlight the double labeling of neurobiotin and ZsGreen, indicating that the cells stained with neurobiotin and previously recorded were those that incorporated ZsGreen from the SVZ. E. Schematic representation of the morphometric Scholl analysis performed on one of the recorded neurons with a series of concentric spheres centered about the cell body at 50 mm intervals. Each of the different dendrites is shown in a different color. **F.** Dendrogram showing the analysis of each of the dendrites of the neuron represented in **E.** In the last dendrogram we see how the analysis has been carried out, counting the number of segments, nodes and endings of each of the dendrites of the cell.

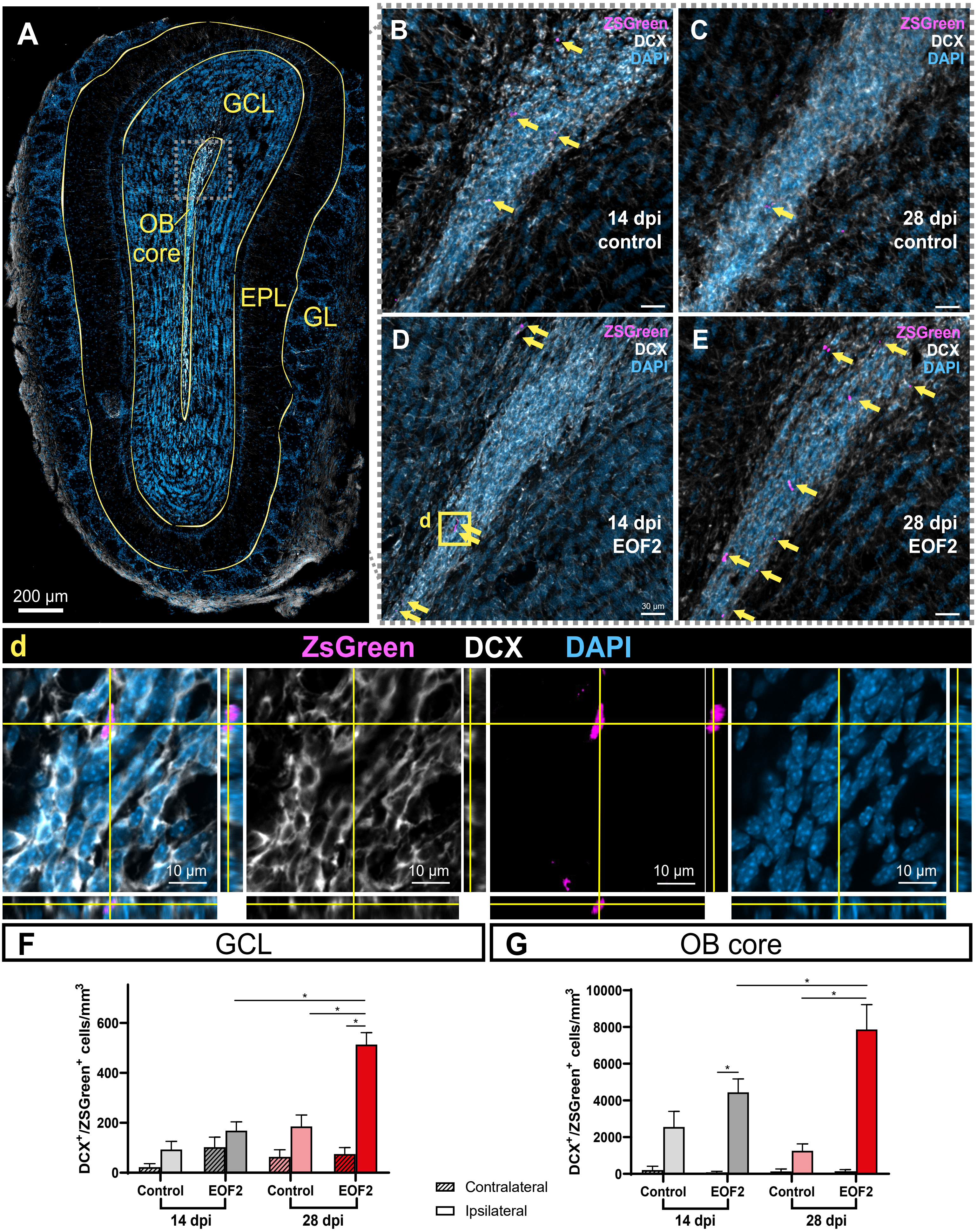

Supplementary Figure S2. Migration of SVZ neuroblast toward the olfactory bulb (OB) increases after a cortical brain injury, and EOF2 enhances neuroblasts migration. **A.** Representative confocal image of a coronal section of the ipsilateral OB of a mice bearing a cortical brain injury. Immunohistochemistry was performed for the detection of the neuroblast marker doublecortin (DCX), nuclear marker DAPI and ZsGreen (not shown). A lentiviral vector expressing ZsGreen was injected in the lateral ventricle ipsilateral to the injury to mark SVZ cells in the same surgical procedure of the injury. Yellow lines delimit the structures of the OB. Dashed grey lines mark the area of the OB core quantified. GCL = granular cell layer; EPL = external plexiform layer; GL = granular layer. **B-E.** Confocal images showing magnification of the outlined area in A for 14 dpi control (B), 28 dpi control (C), 14 dpi EOF2 (D) and 28 dpi EOF2 (E). Contralateral OB not shown. Yellow arrows show ZsGreen+ /DCX+ /DAPI+ cells. Yellow square delimits the area shown in d. ZsGreen is shown in magenta to enhance its visibility. **d.** Orthogonal view of the area marked in D. Yellow lines indicate colocalization of ZsGreen (magenta), DCX (white) and DAPI (blue). Scale bars represent 10 µm. **F.** Quantification of DCX+/ZsGreen+ cells/mm³ in the contralateral and ipsilateral GCL of the OB related to a cortical brain injury in mice treated with vehicle (control) or EOF2 14 days post injury (dpi) or 28 dpi. Data show the mean ±S.E.M. Statistical analysis: * p<0.0001 in two-way ANOVA. **G.** Quantification of DCX+/ZsGreen+ cells/mm³ in the contralateral and ipsilateral OB core related to a cortical brain injury in mice treated with vehicle (control) or EOF2 14 dpi or 28 dpi. Data show the mean ±S.E.M. Statistical analysis: * p<0.0001 in two-way ANOVA.

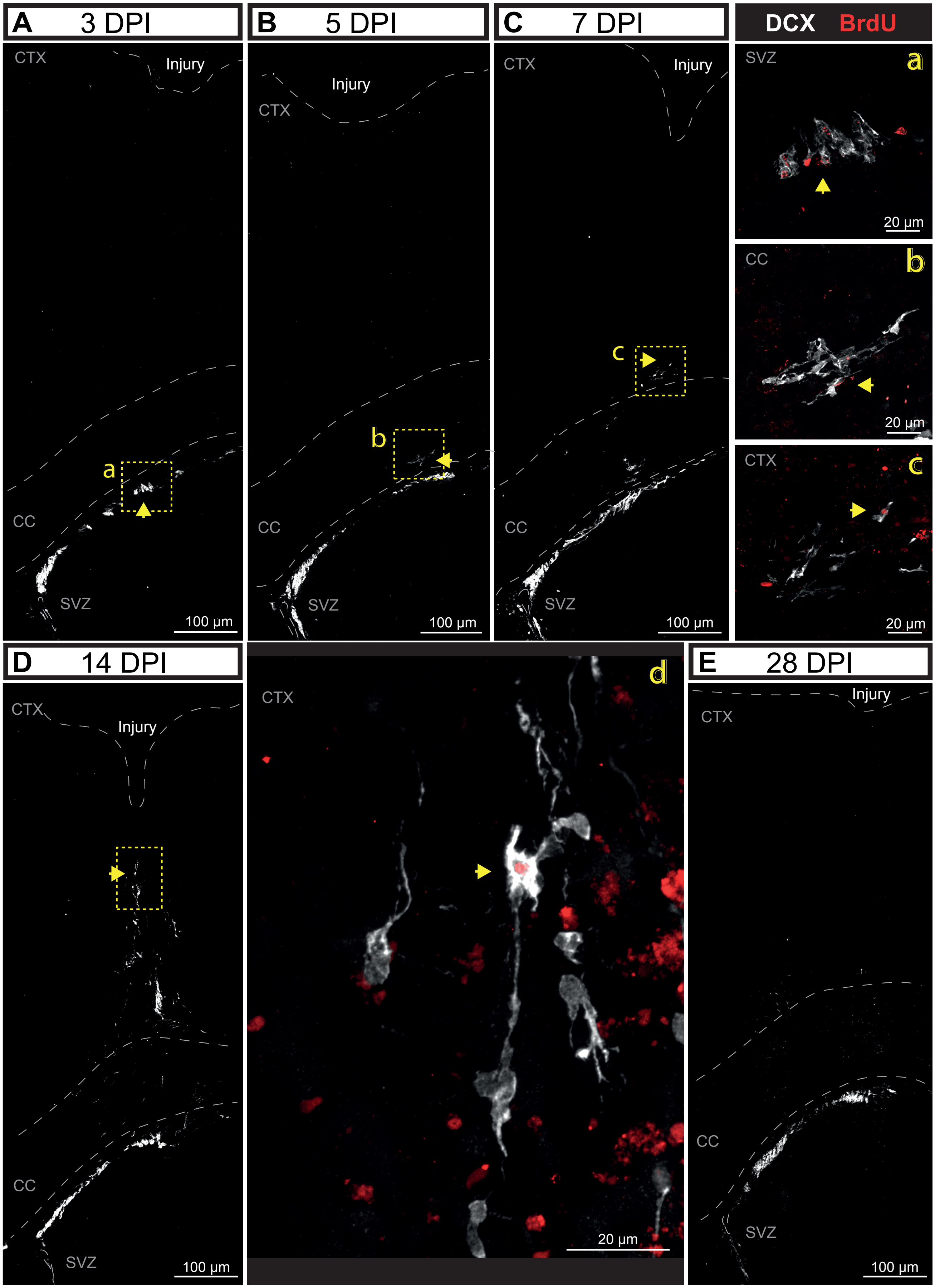

Supplementary Figure S3. EOF2 administration promotes migration of neuroblast to the injured area over time. Mice were mechanically injured in the primary motor cortex and afterwards treated with EOF2 (5 µM). A-E. Representative confocal images of the injured cortex treated with EOF2 showing the immunodetection of DCX at 3 dpi (A), at 5 dpi (B), at 7 dpi (C), at 14 dpi (D) and at 28 dpi (days post-injury) (E), scale bar represents 100 µm. a-d. Magnification of cells indicated with the yellow arrows showing DCX and BrdU (scale bar represents 20 µm). The dotted line indicates the limit of the injury, the CC (corpus callosum) and the limits of the SVZ (subventricular zone). CTX = cortex. 6 animals per group were used for each experiment.

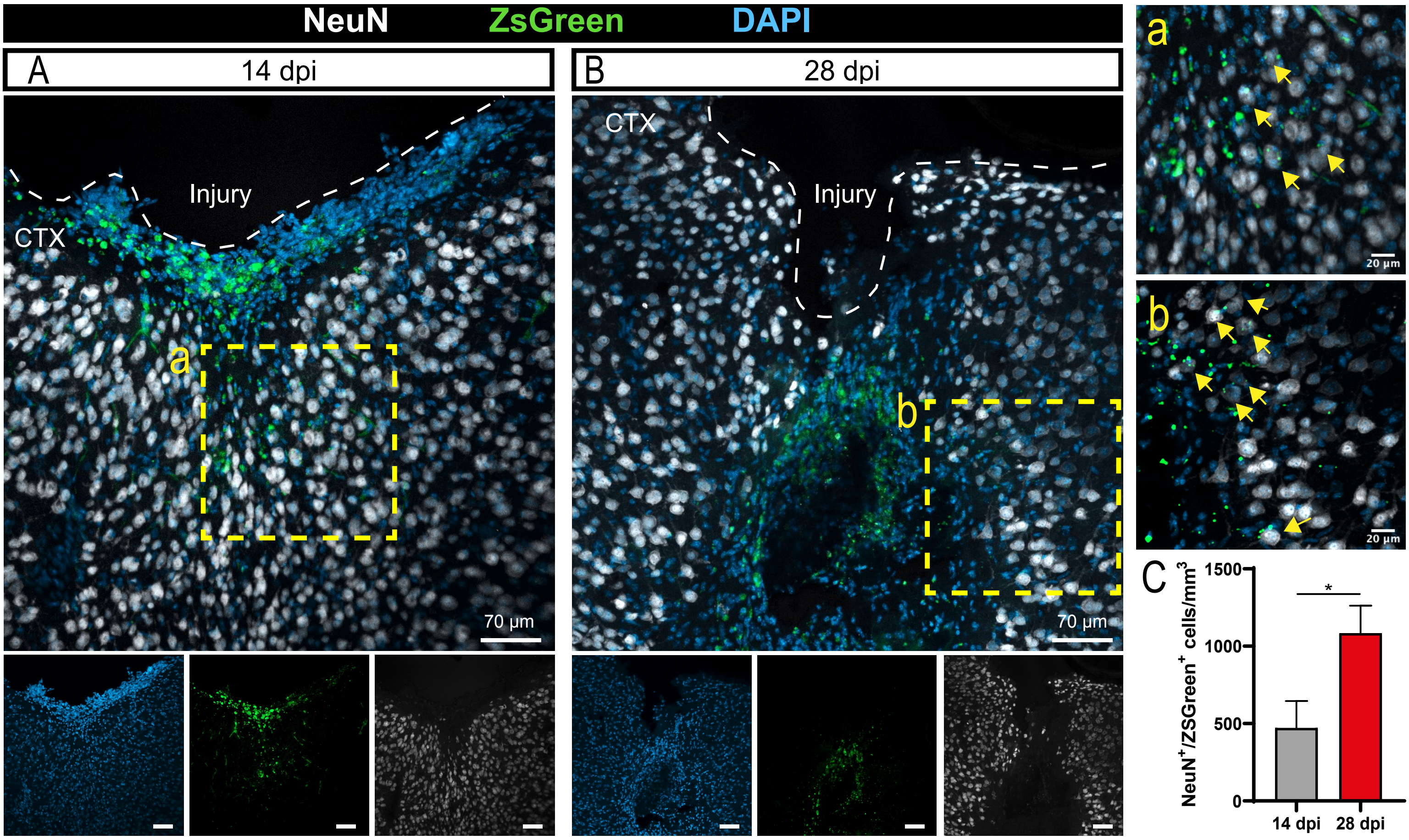

Supplementary figure S4. The number of neurons derived from SVZ cells increases with time in mice treated with EOF2. A-B. Representative confocal images of adult mice cortex bearing mechanical lesions after the administration of EOF2 for 14- (A) or 28- (B) days post injury (dpi). Immunohistochemistry was performed for the detection of nuclear marker DAPI, the neuronal marker NeuN and ZsGreen. A lentiviral vector expressing ZsGreen was injected in the lateral ventricle ipsilateral to the injury to label SVZ cells in the same surgical procedure in which the injury was performed. Scale bars represent 70 µm. a and b show magnifications of the area contained inside the yellow squares. Yellow arrows point at double labeled $ZsGreen^+/NeuN^+$ cells. C. Quantification of double labelled cells $NeuN^+/ZsGreen^+$ per mm3 in the perilesional area at 14 and 28 dpi in mice treated with EOF2. Data show mean ± S.E.M. Statistical analysis: * p= 0.0246 in Student's t-test for equal variance unpaired samples.

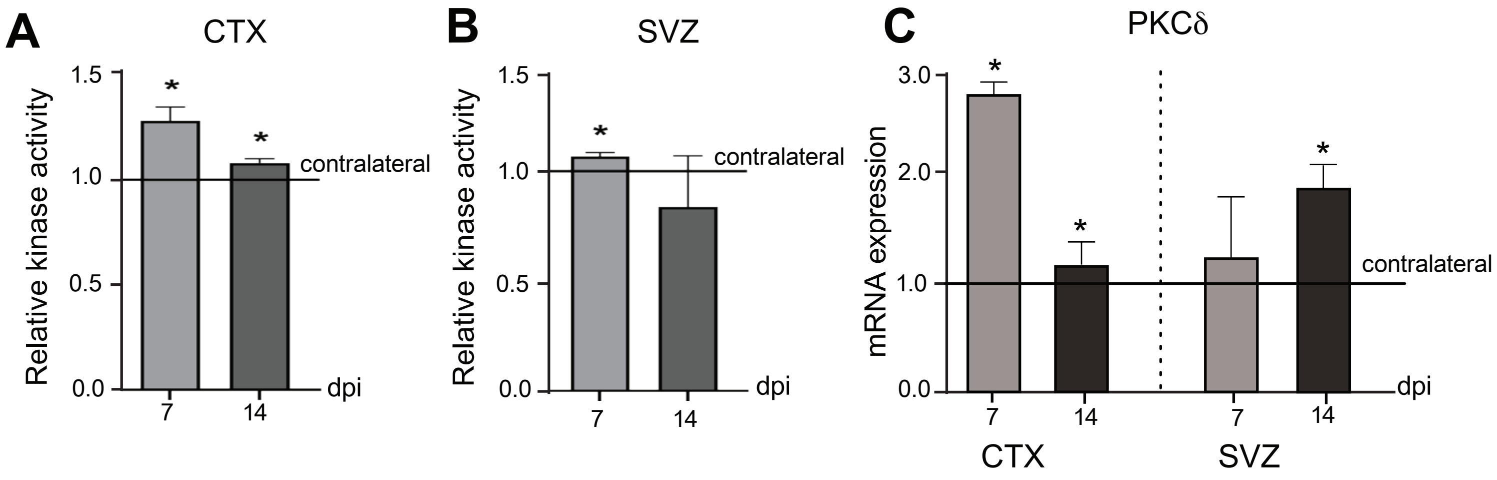

Supplementary Figure S5. PKC activity and PKC   expression in the SVZ and cortex of injured mice. Mechanical cortical lesions were unilaterally performed in the adult mouse primary motor cortex (CTX). Mice were sacrificed 7- or 14-days post injury (dpi). A. Relative protein kinase C activity in the injured cortex (ipsilateral) relative to the non-injured cortex (contralateral) at 7 dpi and at 14 dpi. B. Protein kinase C activity in the injured SVZ (ipsilateral) relative to the non-injured SVZ (contralateral) C. Relative mRNA expression of PKC delta (PKCδ) in the injured cortex (ipsilateral) relative to the non-injured cortex (contralateral) and in the injured SVZ (ipsilateral) relative to the non-injured (contralateral) SVZ at 7 dpi and at 14 dpi. mRNA expression was measured using real time qPCR. All data shown are the means ± SEM of 6 animals per group. *P<0.05.

Supplementary table S1. Primary antibodies table. List of primary antibodies used in the study. Specifying host, isotype, dilution used, epitope retrieval, staining pattern, source and reference.

Antibody	Host	Isotype	Dilution	Epitope retrieval	Staining pattern	Source	Reference
Anti-GFAP	Chicken	Polyclonal	1:1000	GFAP, glial marker	Cytoplasmic	Abcam (Cambridge, UK)	ab4674
	Rabbit	Polyclonal	1:3000			Dako (Santa Clara, CA, USA)	Z0334
Anti-DCX	Rabbit	Polyclonal	1:750	DCX, neuroblast marker	Cytoplasmic	Abcam (Cambridge, UK)	ab18723
Anti-NeuN	Rabbit	Monoclonal	1:500	NeuN, Neuronal marker	Nuclear	Abcam (Cambridge, UK)	ab177487
Anti-Iba1	Rabbit	Monoclonal	1:500	Microglial marker	Nuclear	Abcam (Cambridge, UK)	ab178846
Anti-Nestin	Rabbit	Polyclonal	1:200	Nestin, Neural Stem Cell marker	Cytoplasmic	Abcam (Cambridge, UK)	ab92391
Anti-NRG1	Mouse	Monoclonal	1:500	Neuregulin 1 marker	Cytoplasmic	Invitrogen (Carlsbad, CA, USA)	MA5-12896
Anti-β-III-tubulin	Mouse	Monoclonal	1:1000	β-III-tubulin, neuronal marker	Cytoplasmic	STEMCELL Technologie s (Vancouver, Canada)	60052
Anti-BrdU	Rat	Monoclonal	1:500	BrdU, cell proliferatio n marker	Nuclear	Abcam (Cambridge, UK)	ab6362
Anti-biotin Texas Red conjugated	Goat	Polyclonal	1:900	Biotin (tracer)	Cytoplasmic	Rockland (Pennsylvania, USA)	700-109-098

Supplementary table S2. Secondary antibodies table. List of secondary antibodies used in the study. Specifying host, dilution used, epitope retrieval, staining pattern, source and reference.

Antibody	Host	Dilution	Fluorescence	Source	Reference
Alexa Fluor anti-chicken	Goat	1:1000	647	Abcam (Cambridge, UK)	ab150175
Alexa Fluor anti-rabbit	Donkey	1:1000	647	Invitrogen (Carlsbad, CA, USA)	A32795
Alexa Fluor anti-rabbit	Donkey	1:1000	488	Invitrogen (Carlsbad, CA, USA)	A-21206
Alexa Fluor anti-mouse	Donkey	1:1000	594	Invitrogen (Carlsbad, CA, USA)	A21207
Alexa Fluor anti-rat	Donkey	1:1000	594	Invitrogen (Carlsbad, CA, USA)	A21209

Supplementary table S3. DNA staining. DBA-intercalating-dye used to stain cell nuclei. Specifying target, staining pattern, source and reference.

Intercalating agent	Target	Staining pattern	Source	Reference
DAPI	Double-stranded DNA binding dye	Nuclear	Sigma (St. Louis, MO, USA)	D9542

Supplementary table S4. Intrinsic membrane properties of newly generated neurons from subventricular zone (SVZ).

Current Clamp parameters	7 - 14 dpi	15 - 28 dpi	29 - 56 dpi	57-90 dpi	Pyramidal neurons
Capacitance (pF)	13.7± 1.2+ (n=16)	21.5± 1.0 *+ (n=60)	25.9 ± 1.4 * (n=56)	27.4 ± 1.0 (n=51)	27.6 ± 1.4 (n=38)
Resting Membrane Potential (mV)	-25.1 ± 3.4+ (n=17)	-38.9 ± 2.5 * + (n=58)	-49.1 ± 2.0 * + (n=61)	-60.6 ± 1.4 * (n=50)	-67.8 ± 0.9 (n=38)
Input Resistance (MΩ)	728.0 ± 217.9 + (n=8)	358.7 ± 68.5 * (n=33)	241.5 ± 53.3 (n=29)	168.7 ± 16.3 (n=25)	168.5 ± 10.8 (n=27)
Rheobase (pA)	-	-	126.0 ± 15.7+ (n=15)	205 .0± 19.9 * (n=18)	188.7 ± 10.5 (n=23)
Voltage Threshold (mV)	-	-	-47.2 ± 1.9 (n=18)	-47.6 ± 1.4 (n=21)	-45.6 ± 1.4 (n=36)
Voltage Depolarization (mV)	-	-	12.6 ± 1.3+ (n=17)	18.1 ± 1.1 * (n=19)	23.4 ± 1.3 *+ (n=36)
AP Amplitude (mV)	-	-	56.8 ± 6.6 + (n=14)	76.3 ± 4.7 * (n=24)	90.1 ± 1.6 *+ (n=34)
AP Duration (ms)	-	-	2.2 ± 0.3+ (n=16)	1.7 ± 0.1* (n=20)	1.6 ± 0.1 (n=33)
Maximun Frequency (AP. s^{-1})			10.3±6.1 (4)	15.1±5.3 (7)	21.8±4.2 (21)

The statistical test used was a repeated measures ANOVA followed by a Bonferroni post-hoc test. An asterisk indicates significant differences between two consecutive groups, while a cross indicates significant differences between that group and the group at 57-90 days post-injury (dpi). The significance level was established as p ≤ 0.05. All data are presented as mean ± standard error of the mean. AP, action potential.

Supplementary table S5. Spontaneous synaptic currents and voltage-dependent currents in cortical newly generated neurons from the subventricular zone (SVZ).

Voltage Clamp parameters	7 - 14 dpi	15 - 28 dpi	29 - 56 dpi	57-90 dpi	Pyramidal neurons
Synaptic Frequency (Hz)	-	6.1 ± 1.8+ (10)	4.2 ± 1.0 (18)	1.9 ± 0.3 (23)	1.8 ± 0.5 (5)
Synaptic Amplitude (pA)	-	-39.6 ± 3.4(10)	-39.9 ± 3.2 (18)	-38.7 ± 3.1 (23)	-46.6 ± 4.6 (5)
Outward current density. pA/pF1	7.2 ± 2.1+ (2)	100.5 ± 24.8* (6)	102.6 ± 15.3 (8)	170.2 ± 30.7 (8)	169.3 ± 11.3 (14)
Outward conductance. nS1	0.5 ± 0.1+(2)	17.7 ± 0.8*+ (2)	19.7 ± 2.5 (8)	28.0 ± 2.4 (8)	28.8 ± 1.7 (14)
Inward current density. pA/ pF2	-	-	-31.8 ± 8.1 + (4)	-179.1 ± 18.1* (4)	-281.3 ± 21.4*+ (14)
Inward conductance. nS2	-	-	6.6 ± 3.4+ (4)	21.5 ± 3.7* (4)	34.6 ± 1.9*+ (14)

The statistical test used was a repeated measures ANOVA followed by a Bonferroni post-hoc test. An asterisk indicates significant differences between two consecutive groups, while a cross indicates significant differences between that group and the group at 57-90 days post-injury (dpi). The significance level was established as p ≤ 0.05. All data are presented as mean ± standard error of the mean.

Supplementary table S6. Morphological properties of newly generated neurons from subventricular zone (SVZ).

Morphometric properties	7 - 14 dpi (n=6)	15 - 28 dpi (n=10)	29 - 56 dpi (n=11)	57-90 dpi (n=6)	Pyramidal neurons (n=7)
Somatic Surface Area (μm^2)	244.7 ± 19.0+	449.1 ± 55.1	665.5 ± 108.3	595.8 ± 66.2	660.3 ± 58.0
Dendrites	2.5 ± 0.3+	4.1 ± 0.5+	7.4 ± 0.5 *	7.8 ± 0.3	6.7 ± 0.4
Terminal Endings	2.7 ± 0.3+	9.9 ± 1.3 +	24.1 ± 2.6 *	22.5 ± 2.4	26.3 ± 4.3
Total Dendritic Length (μm)	81.8 ± 47.02+	687.6 ± 134.7+	2506.1 ± 573.6 *	2720.7 ± 445.9	2550.0 ± 800.5
Dendritic Surface Area(μm^2)	231.5 ± 71.2+	2845.9 ± 691.7+	10455.5 ± 2918.6	10651.5 ± 1870.2	9626.7 ± 3222.0
Order	1.2 ± 0.2+	4.0 ± 0.5	5.8 ± 0.9	6.0± 1.5	6.0 ± 0.9
Nº of Nodes	0.2+ 0.2	5.6 ± 1.1+	15.9 ± 2.4 *	14.3 ± 2.2	18.9 ± 3.9

The statistical test used was a repeated measures ANOVA followed by a Bonferroni post-hoc test. An asterisk indicates significant differences between two consecutive groups, while a cross indicates significant differences between that group and the group at 57-90 days post-injury (dpi). The significance level was established as p ≤ 0.05. All data are presented as mean ± standard error of the mean.

Supplementary table S7. Morphofunctional properties of newly generated neurons from subventricular zone (SVZ) based on cortical layer integration.

Morphofunctional properties	Neurons from upper layers (I-IV) (n=8)	Neurons from botton Layers (V-VI) (n=9)
Total Surface Area (μm^2)	7350 ± 1513.2	14557.4 ± 3264.9*
Total Dendritic Length (μm)	1599.0 ±354.2	3456.4 ± 0.530.1*
Total Segments	29.0 ± 2.5	47.7 ± 4.6 *
Terminal Endings	18.2 ± 1.2	28.5 ± 2.6*
Rheobase (pA)	113.8 ± 20.8	190.0 ± 28.8*
Input Resistance (MΩ)	146.8 ± 20.6	123.4 ± 15.0
Frecuency Gain (AP.s^{-1}.nA^{-1})	433.1± 185.6	55.4 ± 10.0*

The statistical test used was a Student's t-test. Differences between the two groups are indicated with an asterisk. The significance level was established as p ≤ 0.05. All data are presented as mean ± standard error of the mean. AP, action potential
